# Development of a Gas Chromatography-Time-of-Flight Method for Detecting Glucosinolate Metabolites and Volatile Organic Compounds in Kimchi

**DOI:** 10.1155/2021/9978251

**Published:** 2021-06-18

**Authors:** Ho Jin Kim, Mi Jin Lee, Suel Hye Hur, Min Hee Jeong

**Affiliations:** National Agricultural Products Quality Management Service, Gimcheon 39660, Republic of Korea

## Abstract

This study examined the volatile organic compounds (VOCs) and metabolites of glucosinolate (GLS) contained in kimchi and analyzed GLS using myrosinase. The analysis was conducted using gas chromatography-time of flight (GC-TOF), and VOC and the metabolite quantities were detected and analyzed. Based on 22 samples, tests were conducted, and 12 metabolites and 52 VOCs were found. When the detected metabolites were compared in general, the rate of isothiocyanate, which is well known for its anticancer effects and various other activities, was the highest. A total of 52 VOCs, including 15 aliphatic hydrocarbons, 7 acids, and 6 alcohols, were detected by GC-TOF. Therefore, the analytical methods provide a good basis to examine VOC and GLS metabolites; furthermore, the methods are of great help to secure excellent kimchi and evaluate its quality.

## 1. Introduction

Traditional fermented food, kimchi, was certified by Codex Alimentarius in July 2001 and is recognized as a global dish that is distinct from other types of pickles [1]. It is made by adding various ingredients (chili pepper powder, garlic, ginger, spring onion, radish, etc.) and salted seafood to cabbage, the main ingredient, which is then fermented at low temperature based on lactic acid bacteria [2–4]. The unique flavor of kimchi is derived from the ingredients and produced during the fermentation process. Kimchi is rich in vitamins and minerals, and it is a low-calorie food that plays an important role in reducing cholesterol. One of the effects of kimchi is its anticancer effect, which is promoted by minor ingredients of kimchi, namely, garlic and dried red pepper powder [5].

**Figure 1:**
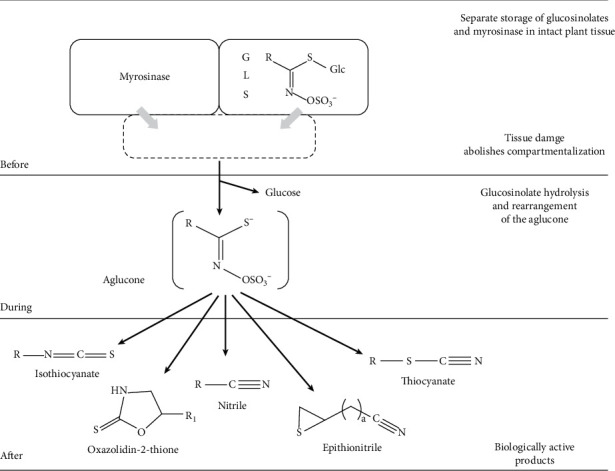
Functional and biological implications of the myrosinase-glucosinolate system.

Kimchi contains glucosinolate (GLS), which is one of the main components of Cruciferae, metabolites, and volatile organic compounds (VOCs). First, GLS and its decomposition products are known for excellent effects. As shown in Figure 1, GLS decomposes into isothiocyanate (a bioactive substance), nitrile (a toxic compound), thiocyanate, oxazolidine-2-thiones, and thiocyanates, as well as epithionitriles and glucosevia, the myrosinase enzyme [6].

Isothiocyanates are involved in the defense mechanism of plants, offering strong antibacterial and insecticidal attributes, as well as strong flavor; isothiocyanates also help prevent cancers, such as liver, lung, and stomach cancers in human beings [7, 8]. Particularly, sulforaphane (the isothiocyanate of glucoraphanin) and indole-3-carbinol (the isothiocyanate of glucobrassicin) are excellent contributors to the anticancer effect of kimchi [9–11]; according to experimental and statistical results reported in other studies, the prevalence of breast cancer can be reduced with the consumption of crucifers [12]. In animal testing, tumors causing skin cancer were suppressed by increasing consumption of crucifers [13]. Furthermore, sulforaphane reduces the size and occurrence of tumors, as well as delays their growth [14]. Sulforaphane is also known to promote defense mechanisms against insects and pathogens and to be involved in sulfur, nitrogen, and glucose metabolism [15]. In addition, sulforaphane influences hormone auxin levels, and it influences colon, lung, and prostate cancers in human beings with anticancer compounds [16]. Many pharmacological and physiological activities have been reported, among many other research studies on these compounds in kimchi [9–11]. Accordingly, for GLS, various studies have been conducted on the VOC composition and discrimination of GLS decomposition products in crucifers [17]; one study on GLS in serum, using LC-MS/MS and its decomposition products, was also completed [18]. However, studies on GLS metabolites are lacking.

VOCs are known to have the most powerful effects on the sourness, organoleptic properties, and palatability of kimchi [19]. There are various types of VOCs, such as dimethyl sulfides, sulfide, isothiocyanates, aldehydes, ketones, and alcohols. In relevant research, 25 VOCs collected by SPME [20], 40 types of sulfides [21], and GLS-derived VOCs [6] were identified.

Many analytical instruments have been recently developed to provide many advantages, including decreasing analysis time, saving sample and extraction solvent amounts, detecting minute amounts with high sensitivity, separating mixtures, identifying structures, and analyzing minute samples. Moreover, the appearance of TOF recently is advantageous to measure the exact mass with high resolution, high sensitivity, and strengthened selectivity [22–25]. Because properties that could not be analyzed before have since become resolvable using these analytical instruments, it has become possible to develop new analytical methods.

This study investigated GLS and VOC metabolites in myrosinase-treated kimchi using GC-TOF. The manufacturers and ingredients of various kimchi samples were used to analyze the GLS metabolites and VOCs. The findings can be used to verify the benefits of kimchi and the use of various cruciferous vegetables.

## 2. Materials and Methods

### 2.1. Samples, Chemicals, and Reagents

Tests were made with various kinds of kimchi purchasable in local marts; a total of 22 samples were used. In the experiment, 22 kimchi were sliced and prefreezed for 48 hours at −40°C. After that, it is freeze-dried in a dryer at −70°C for 24 hours. Freeze-dried kimchi was pulverized using a food-mixer blender and filtered with a 3 mesh size. Water was purified using a Milli-Q® RiOsTM/Elix® water purification system (Millipore, Bedford, MA, USA). Myrosinase enzymes and L-ascorbic acid were purchased from Sigma (St. Louis, MO, USA). Dichloromethane was purchased from Merck KGaA (Darmstadt, Germany). Anhydrous sodium sulfate was purchased from Dae Jung.

### 2.2. Sample Preparation

5 g of the lyophilized sample was placed in a volumetric flask, and 250 mL of distilled water, 5 units of myrosinase enzyme, and 5 mg of L-ascorbic acid were added to the mixture. The mixture was then kept at room temperature for 2 hours. Then, 100 mL of dichloromethane was added and stirred for 30 minutes. The layers were separated by centrifugation at 3,500 rpm for 15 minutes. The separated organic solvent layer was passed through anhydrous sodium sulfate, and the sample was then concentrated in a rotary evaporator until 0.5 mL of the sample remained. After the concentrate temperature reached −20°C, the sample was tested.

### 2.3. GC-TOF Analysis

GC was performed on an HP 7890 GC (Agilent Technologies, Santa Clara, CA, USA), and the injector was used on a rail system (CTC, Gerstel, LEAP). The injection temperature was set at 250°C, and the injection type was set at split 10 : 1 mode. The other experimental parameters were set as follows: carrier gas He, flow rate 1.5 mL/min, column RTX-5 MS 30 m × 0.25 mm × 0.25 *μ*m, and transfer line temperature (260°C).

Detection was performed on a LECO®PegasusHT® TOF-MS and controlled using a Chroma TOF (LECO, St. Joseph, MI, USA). The other experimental parameters were set as follows: acquisition delay 130 s, mass range (5–650 *μ*, acquisition rate 10 spectra/s, detector voltage 1650 V, electron energy 70 eV, and ion source temperature 250°C.

## 3. Results and Discussion

**Figure 2:**
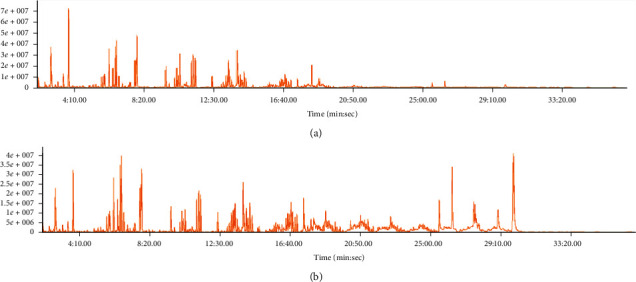
TIC (total ion chromatogram) of samples 17 (a) and 21 (b).

Tests were conducted based on 22 samples purchasable in marts and repeated three times. VOC and metabolites of GLS ingredients were detected in all 22 samples (Figure 2) with an S/N ratio of more than 50. The results are shown in Sections 3.1 and 3.2.

### 3.1. Composition Analysis of Glucosinolate Metabolite in Kimchi

**Figure 3:**
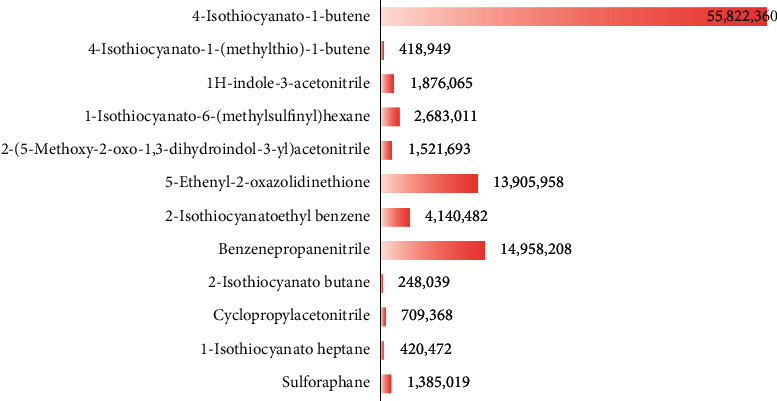
Average area of glucosinolate metabolites in kimchi.

GLS metabolites can be largely divided into the following five types: isothiocyanate, oxazolidin-2-thione, nitrile, epithionitrile, and thiocyanate. In this study, 7 istiocyanates, 1 oxazolidin-2-thione, and 4 nitriles were detected. The 12 GLS metabolites were classified based on the GC library, and the sample area was confirmed. First, there were 7 isothiocyanates including 1-isothiocyanato heptane, 2-isothiocyanato butane, and 2-isothiocyanatoethyl benzene, and sulforaphane was identified as a glucoraphanin metabolite, which is a type of GLS (found in the GLS comparative study). This suggests that samples containing sulforaphane have glucoraphanin. On average, 4-isothiocyanato-1-butene was the largest at 55,822,360, especially in samples 17 and 21, which had values of 229,507,199 and 278,971,384, respectively. In addition, in samples 17 and 21, 2-isothiocyanatoethyl benzene was also found in large quantities of 37,200,818 and 39,458,110, respectively; however, the compound was present in all samples. The average area of oxazolidin-2-thione class metabolites was 13,905,958, and the area was observed only in samples 17 and 21. Among nitrile metabolites, cyclopropylacetonitrile, benzenepropanenitrile, 2-(5-methyoxy-2-oxo-1,3-dihydroindol-3-yl) acetonitrile, and 1H-indole-3-acetonitrile were found. Benzenepropanenitrile was observed in the highest content with an area of 0–39,724,396 (average 14,958,208). More detailed results are presented in Figure 3, Table 1, and Table S1 [20, 21, 26–29].

### 3.2. Composition Analysis of Kimchi VOC

**Figure 4:**
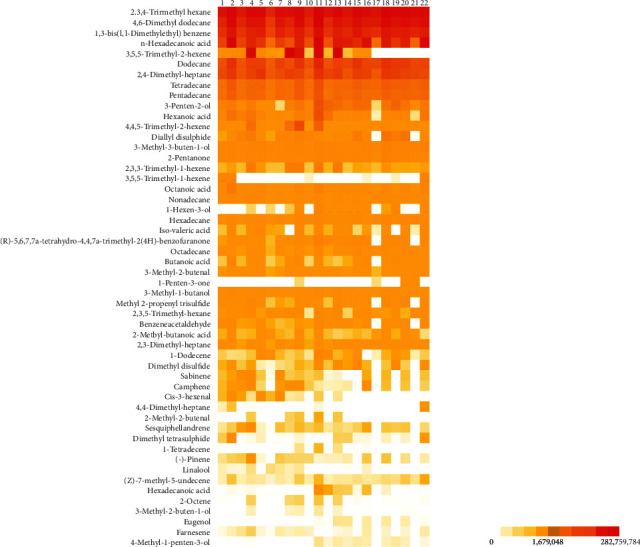
Heat map of volatile organic compounds (VOCs) in kimchi.

Table 2 classifies VOCs detected in the 22 samples according to class. In total, 52 VOCs were observed, specifically aliphatic hydrocarbons (15), acids (7), alcohol (6), alkane (5), ketone (5), aldehyde (4), S-containing compounds (4), terpene hydrocarbons (5), and a miscellaneous compound (1) (Table 2). The largest areas were occupied by 2, 3, 4-trimethyl hexane (214,485,249), 4, 6-dimethyl dodecane (176,577,662), 1,3-bis (1,1-dimethylethyl), and benzene (139,454,474), in that order. A comparison of the sample areas showed that samples 11 and 18 of 2,3,4-trimethyl hexane had the largest areas (275,021,365 and 263,894,944, respectively). Among the 52 compounds, only 21 were observed in all samples, with the average area of 145,926–214,485,249. The overall results are presented in Figure 4 and Table S2. The mass spectrum and structure are shown in Table 2 and Figure S1.

## 4. Conclusions

The cell walls of Brassicaceae plants contain an enzyme called myrosinase. When external animals and insects destroy the cell wall or the cell wall is destroyed by microorganisms or bacteria, an enzyme called mirosinase is released. The enzymes in myrosinase break down GLS, resulting in the production of secondary metabolites. In kimchi, 51 compounds of VOCs and GLS metabolite were detected by GC-TOF. This analysis technique was used for analyzing VOCs and GLS metabolites contained in kimchi, based on myrosinase treatment. For the analysis, GC-TOF was used in comparison with the library and to identify the compounds. As a result, in 22 samples, 12 GLS metabolites and 51 VOCs were observed. The findings of GLS metabolites and the GC-TOF analysis technique in this study can provide useful data for relevant analyses and quality assessments, as well as provide evidence of the excellence and reliability of kimchi.

## Figures and Tables

**Table 1:** Structure of glucosinolate metabolites in kimchi.

	
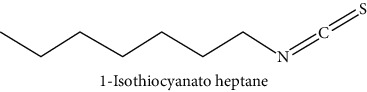	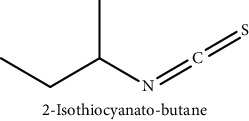
	
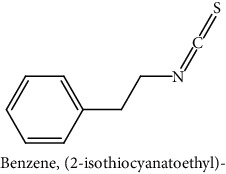	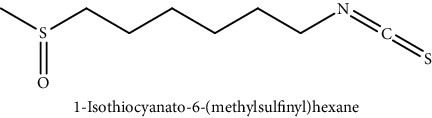
	
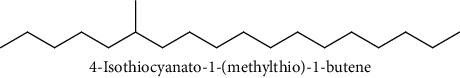	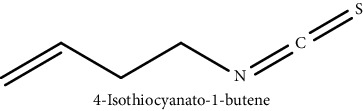
	
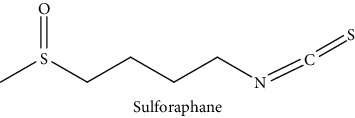	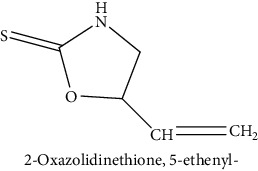
	
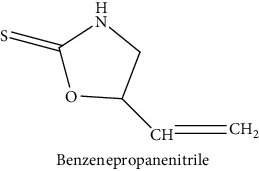	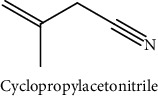
	
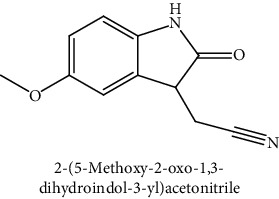	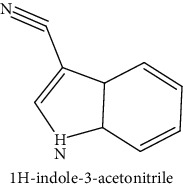
	
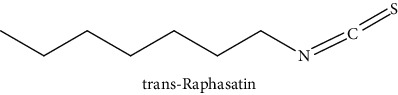	

**Table 2:** Classified VOCs.

NO.	Compound	Chemical group	Reference
1	1-Penten-3-one	Ketone	[26]
2	2-Methyl-2-butenal	Aldehyde	[26]
3	cis-3-Hexenal	Aldehyde	[26]
4	Di-2-propenyltrisulfide,	S-containing compounds	[21]
5	Diallayldisulphide	S-containing compounds	[21]
6	Dimethyl tetrasulphide	S-containing compounds	[21]
7	Farnesene	Terpene hydrocarbons	[21, 28]
8	Sesquiphellandrene	Terpene hydrocarbons	[21, 28]
9	Sabinene	Terpene hydrocarbons	[21, 28]
10	(-)-Pinene	Terpene hydrocarbons	[28]
11	Camphene	Terpene hydrocarbons	[28]
12	2,4-Dimethyl- heptane	Aliphatic hydrocarbons	[28]
13	4,6-Dimethyl- dodecane	Aliphatic hydrocarbons	[28]
14	Tetradecane	Aliphatic hydrocarbons	[28]
15	Hexadecane	Aliphatic hydrocarbons	[28]
16	1,3-bis (1,1-Dimethylethyl)- benzene	Miscellaneous compounds	[28]
17	Butanoic acid	Acids	[27]
18	iso-Valeric acid	Acids	[27]
19	2-Methylbutanoic acid	Acids	[27]
20	n-Hexadecanoic acid	Acids	[27]
21	3-Methyl-1-butanol,	Alcohols	[27]
22	3-Methyl-2-buten-1-ol	Alcohols	[27]
23	Linalool	Alcohols	[27]
24	Eugenol	Alcohols	[27]
25	(R)- 5,6,7,7a-tetrahydro-4,4,7a-trimethyl-2 (4H)-benzofuranone	Ketone	[27]
26	Dodecane	Aliphatic hydrocarbons	[27]
27	1-Dodecane	Aliphatic hydrocarbons	[27]
28	1-Tetradecene	Aliphatic hydrocarbons	[27]
29	Benzeneacetaldehyde	Aldehyde	[29]
30	Nonadecane	Alkane	[29]
31	Octadecane	Alkane	[29]
32	Hexadecanoic acid	Acids	[29]
33	Pentadecane	Alkane	[29]
34	Dimethyl disulfide,	S-containing compounds	[20]
35	Hexanoic acid	Acids	[20]
36	Octanoic acid	Acids	[20]
37	4-Methyl-1-penten-3-ol	Ketone	[26]
38	3-Methyl-2-butenal	Aldehyde	[26]
39	1-Hexen-3-ol	Alcohols	[26]
40	2-Pentanone	Ketone	[26]
41	2,3,5-Trimethyl- hexane	Aliphatic hydrocarbons	[28]
42	2,3,4-Trimethyl- hexane	Aliphatic hydrocarbons	[28]
43	4,4,5-Trimethyl-2-Hexene,	Aliphatic hydrocarbons	[28]
44	3,5,5-Trimethyl-2-Hexene,	Aliphatic hydrocarbons	[28]
45	3,5,5-Trimethyl-1-Hexene,	Aliphatic hydrocarbons	[28]
46	2,3,3-Trimethyl-1-hexene	Aliphatic hydrocarbons	[28]
47	4,4-Dimethyl- heptane,	Aliphatic hydrocarbons	[28]
48	2,3-Dimethyl- heptane,	Aliphatic hydrocarbons	[28]
49	3-Penten-2-ol	Ketone	[27]
50	3-Methyl-3-buten-1-ol	Alcohols	[27]
51	2-Octene	Alkene	[27]

## Data Availability

All data are included within the article and the supplementary materials.

## Supplementary Materials

Supplementary Materials More detailed composition analysis of glucosinolate metabolite and VOC are presented. Table 1: area of glucosinolate metabolites. Table 2: area of volatile oragnic compounds. Figure S1: GC-TOF of mass spectrum and structure of compounds.
