# Supplementary material for: Development of a Gas Chromatography-Time-of-Flight Method for Detecting Glucosinolate Metabolites and Volatile Organic Compounds in Kimchi
Source: Int J Anal Chem. 2021 Jun 18;2021:9978251. doi: 10.1155/2021/9978251 (PMC8233085; doi:10.1155/2021/9978251)
Supplement: Supplementary Materials — More detailed composition analysis of glucosinolate metabolite and VOC are presented. Table 1: area of glucosinolate metabolites. Table 2: area of volatile oragnic compounds. Figure S1: GC-TOF of mass spectrum and structure of compounds. [file 9978251.f1.zip › 9978251.f1/Supplement Fig. 1.pdf]

## 1) 1-Penten-3-one

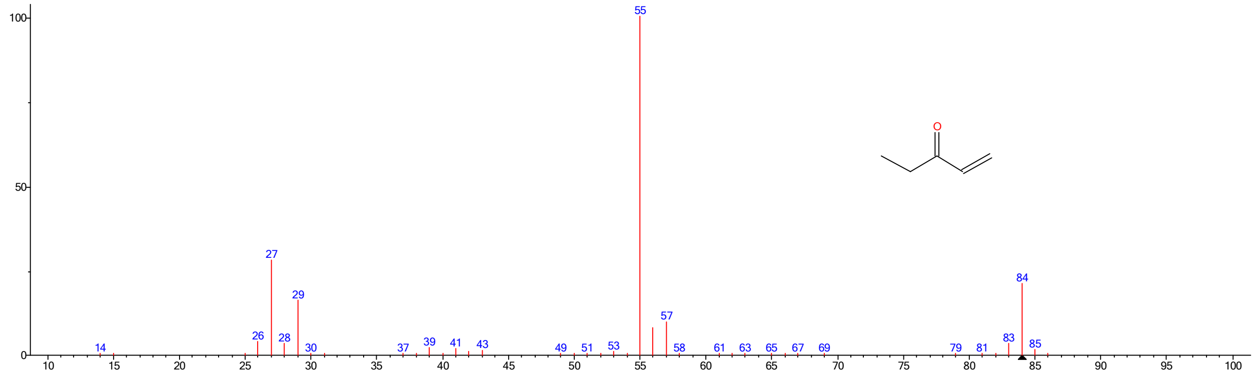


(mainlib) 1-Penten-3-one

## 2) 2-Methyl-2-Butenal

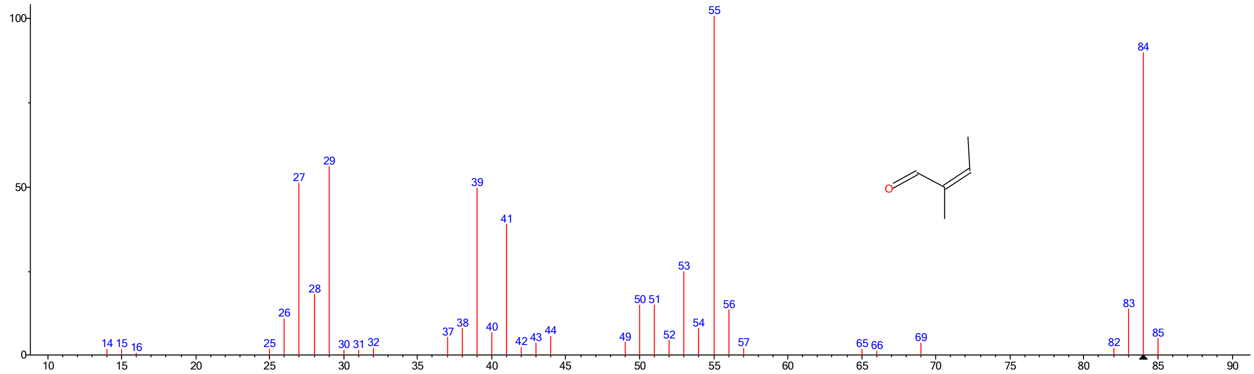


(mainlib) 2-Butenal, 2-methyl-, (E)-

## 3) cis-3-Hexenal

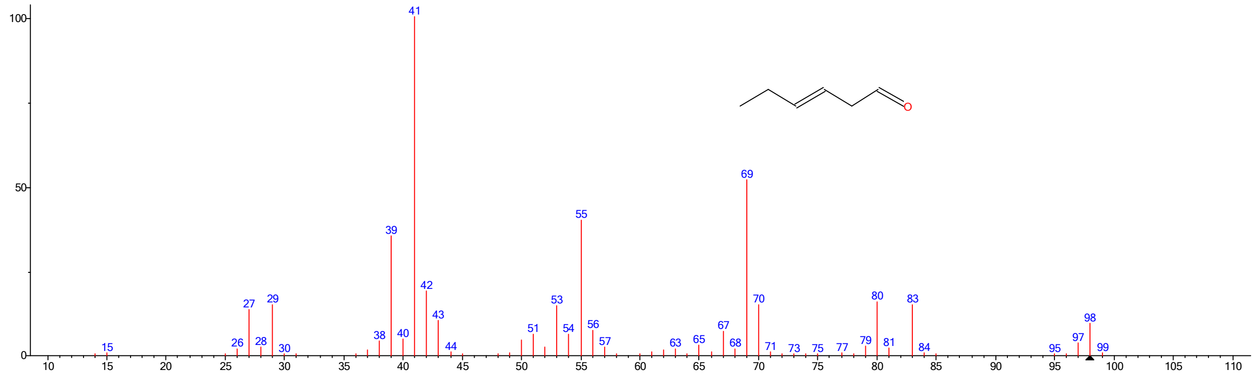


(mainlib) 3-Hexenal

## 4) di-2-propenylTrisulfide (Trisulfide,di-2-propenyl)

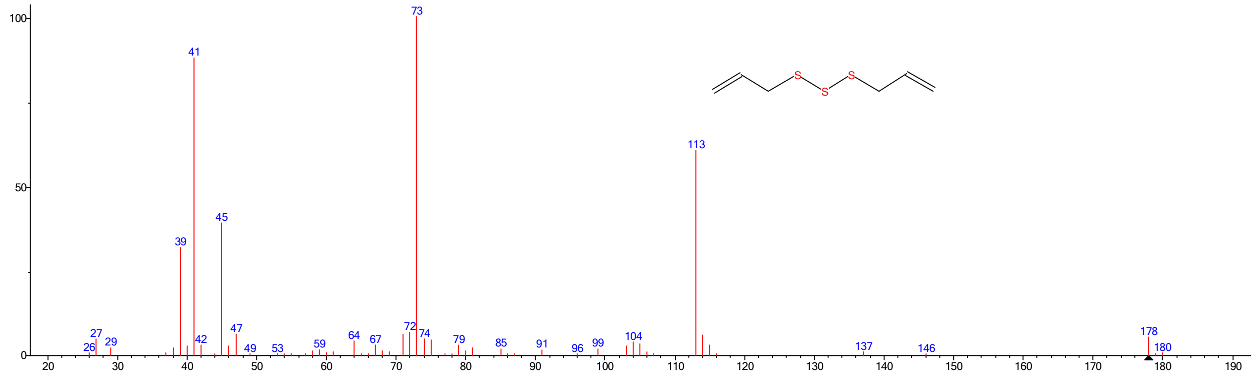


(mainlib) Trisulfide, di-2-propenyl

## 5) Diallayldisulphide (1.2-Di((E)-prop-1-en-1yl)disufane)

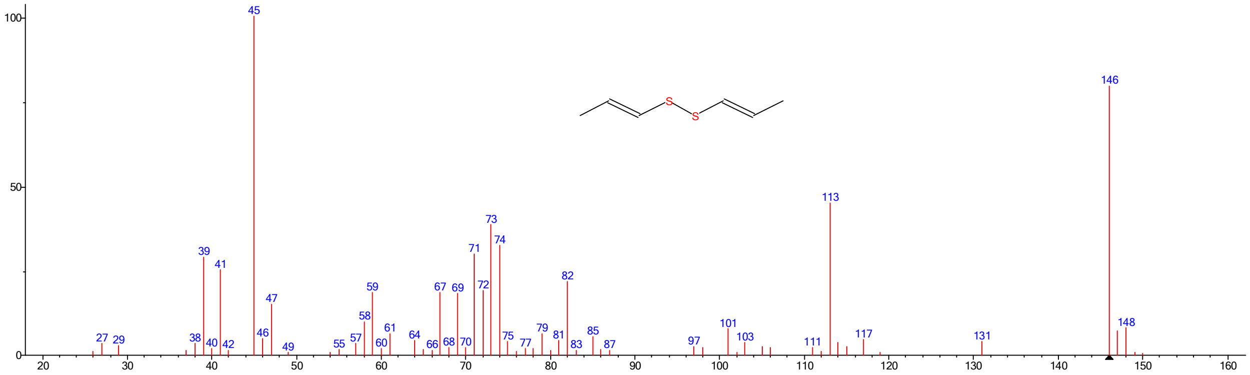


(mainlib) 1,2-Di((E)-prop-1-en-1-yl)disulfane

## 6) Dimethyl tetrasulphide

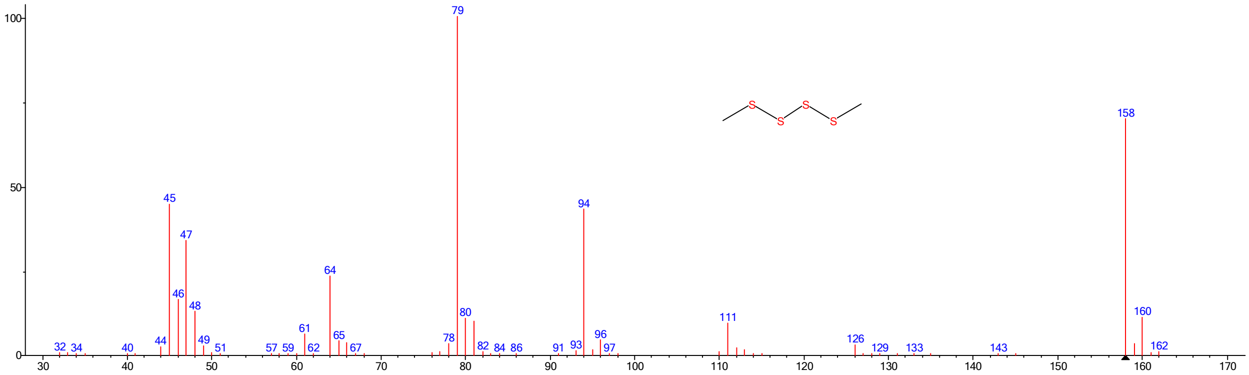


(mainlib) Tetrasulfide, dimethyl

## 7) Farnesene

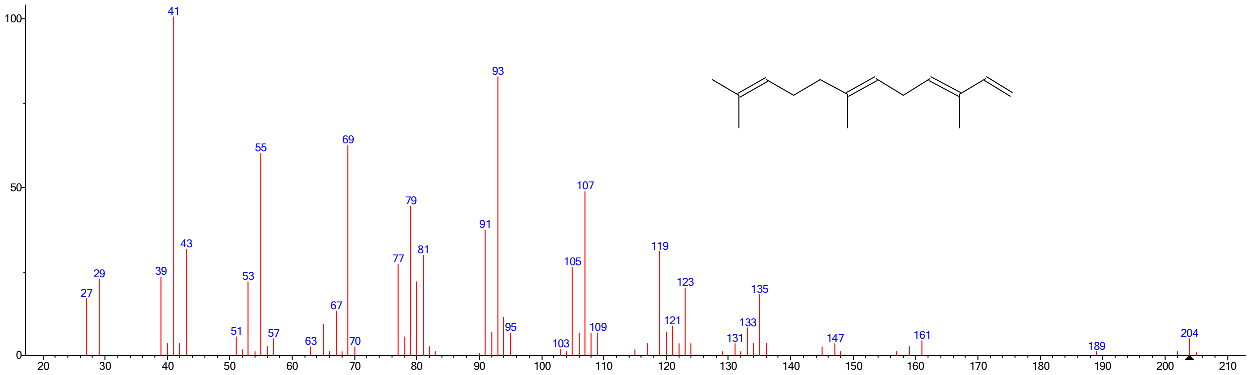

(mainlib) α-Farnesene

## 8) Sesquiphellandrene

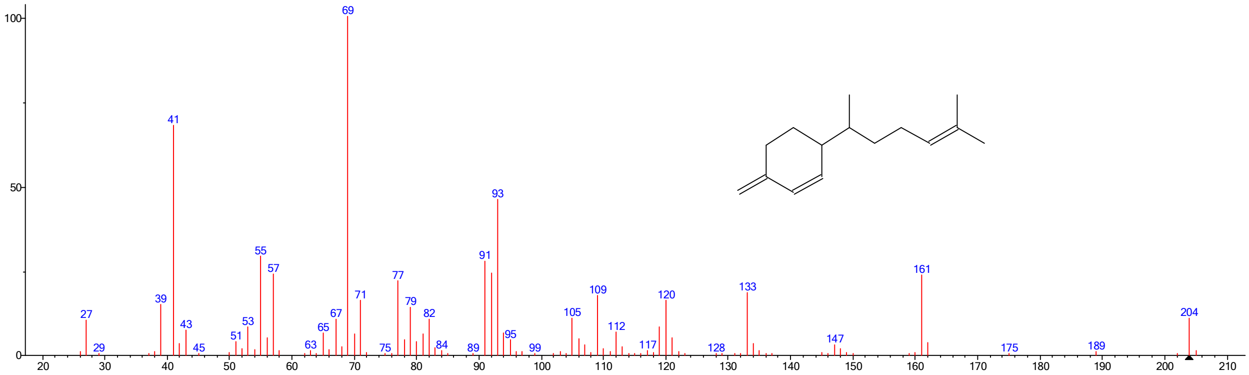

(mainlib) Cyclohexene, 3-(1,5-dimethyl-4-hexenyl)-6-methylene-, [S-(R*,S*)]-

## 9) Sabinene

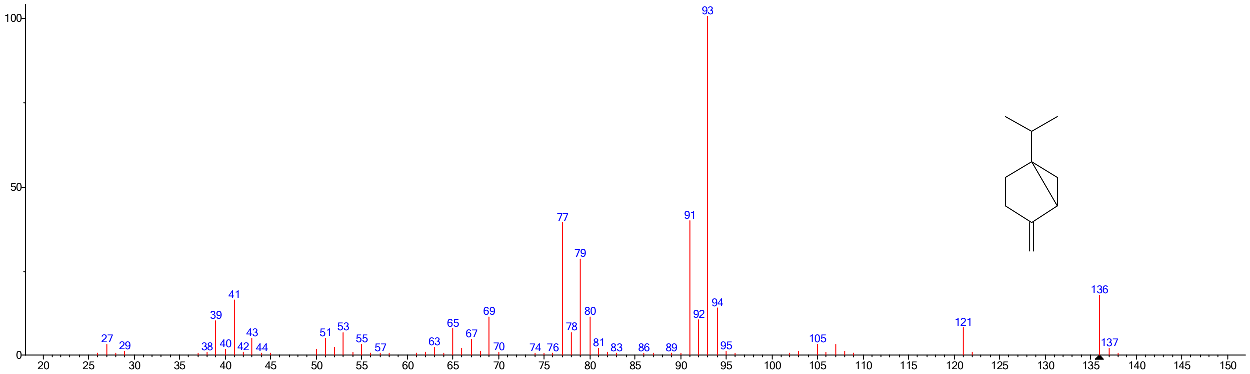


(mainlib) Bicyclo[3.1.0]hexane, 4-methylene-1-(1-methylethyl)-

## 10) (-)-pinene

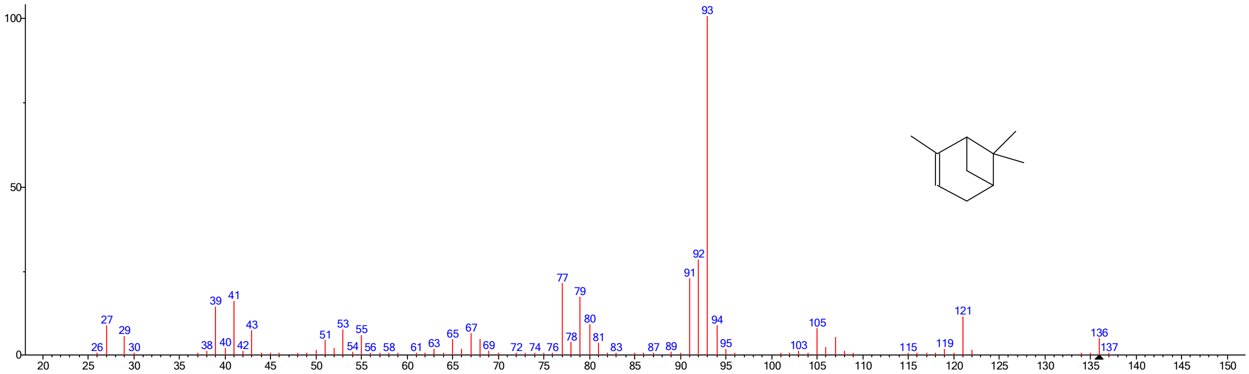


(mainlib) (1S)-2,6,6-Trimethylbicyclo[3.1.1]hept-2-ene

## 11) Camphene

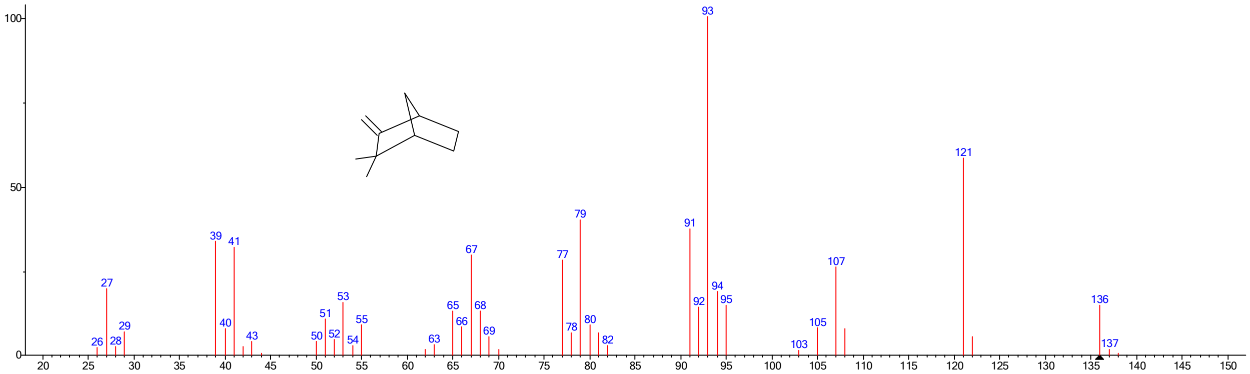


(mainlib) Camphene

## 12) 2,4-dimethyl- Heptane

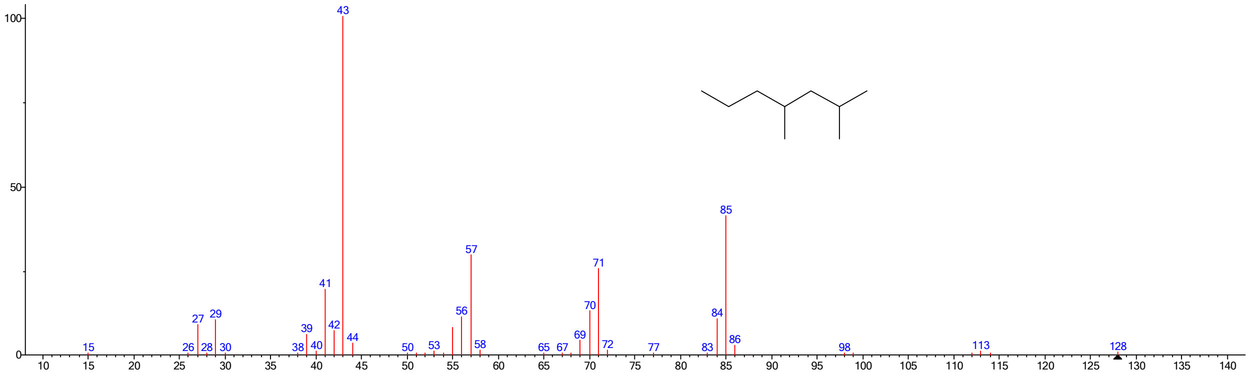


(mainlib) Heptane, 2,4-dimethyl-

## 13) 4,6-dimethyl- Dodecane

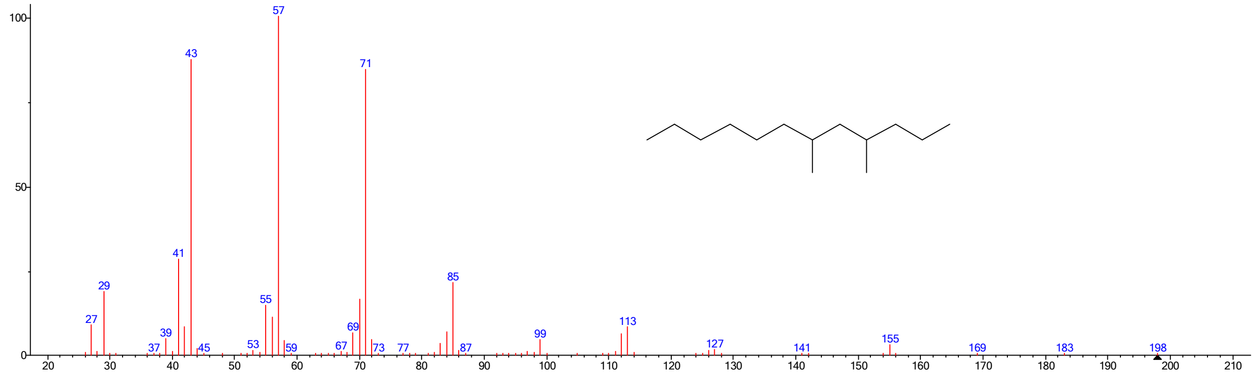


(mainlib) Dodecane, 4,6-dimethyl-

## 14) Tetradecane

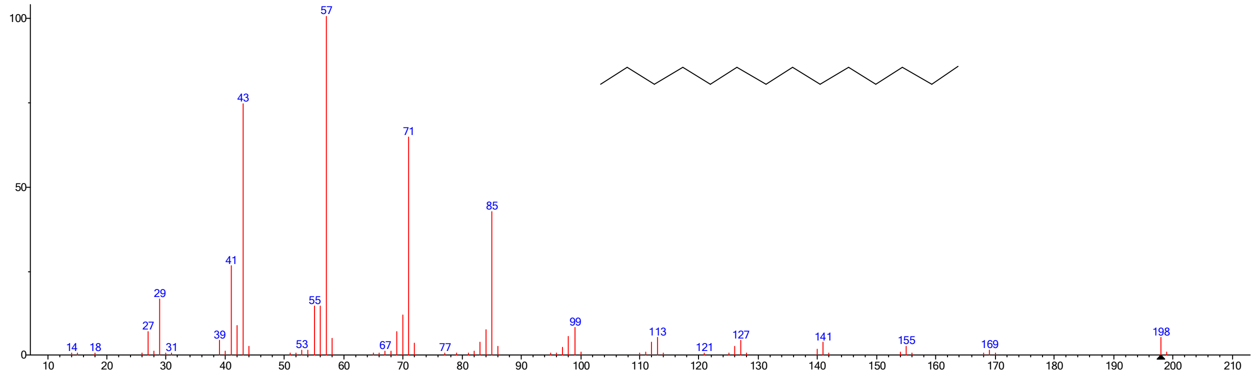


(mainlib) Tetradecane

## 15) Hexadecane

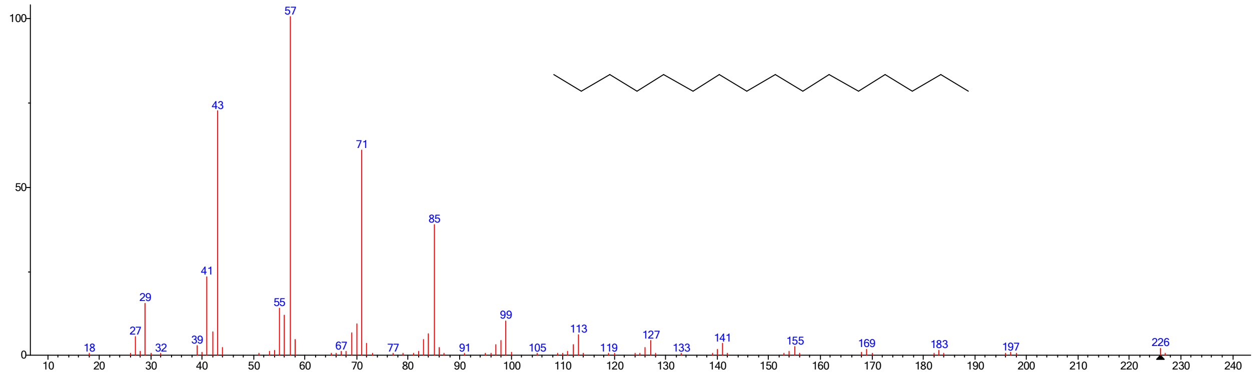


(mainlib) Hexadecane

## 16) 1,3-bis(1,1-dimethylethyl)- Benzene (1.3-bis(1.1dimetylehyl))

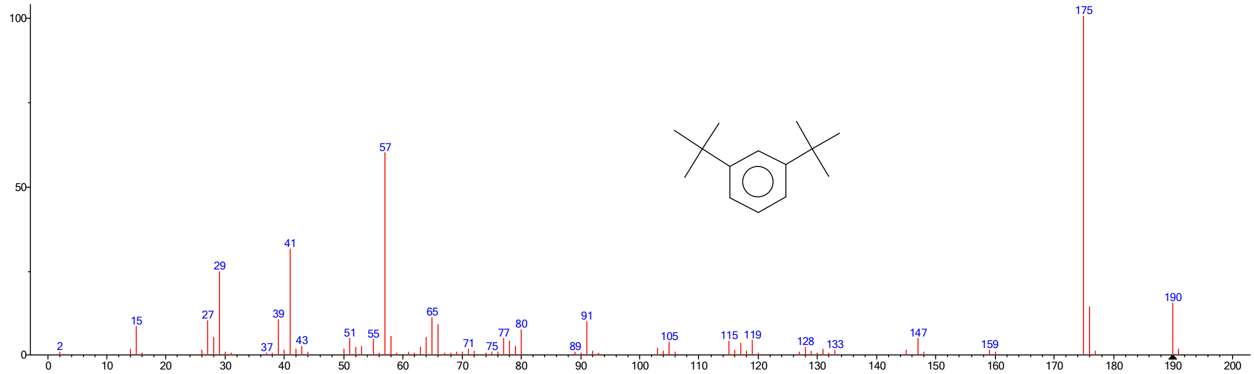


(mainlib) Benzene, 1,3-bis(1,1-dimethylethyl)-

## 17) Butanoic acid

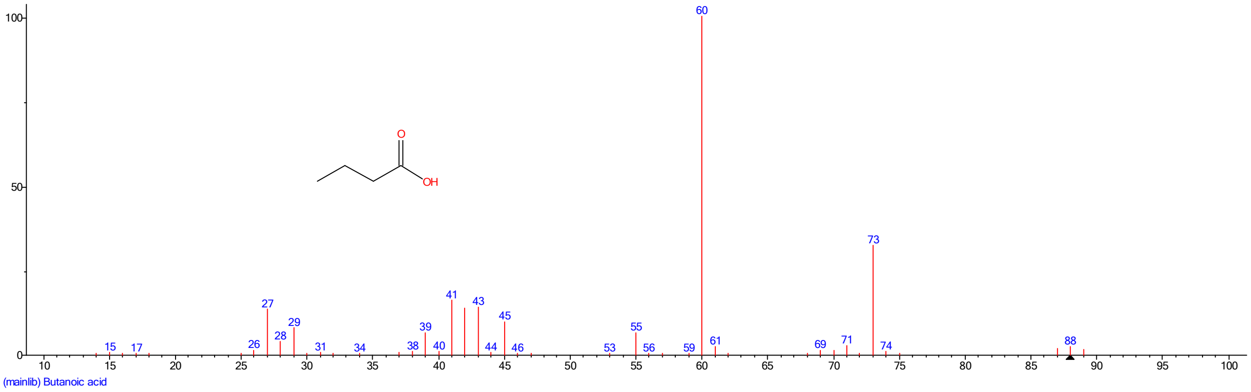


## 18) iso-Valeric acid

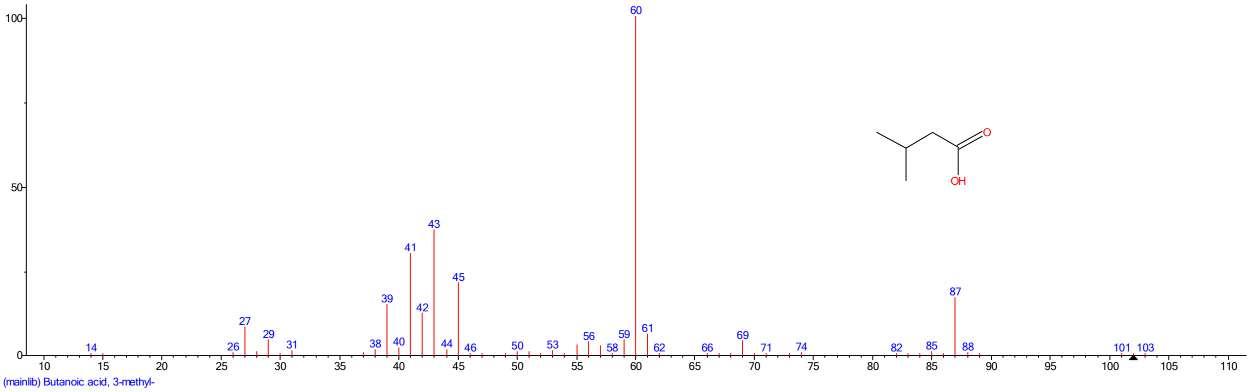


## 19) 2-methylButanoic acid,

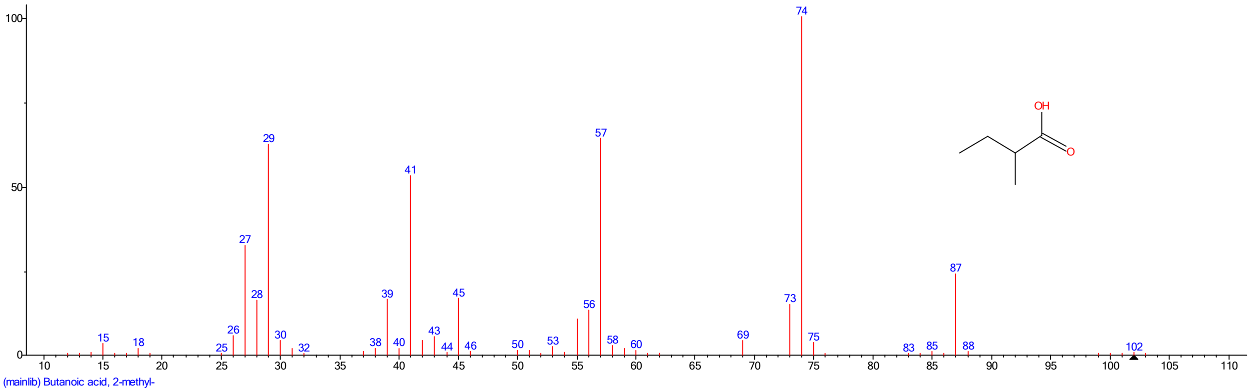


## 20) n-Hexadecanoic acid

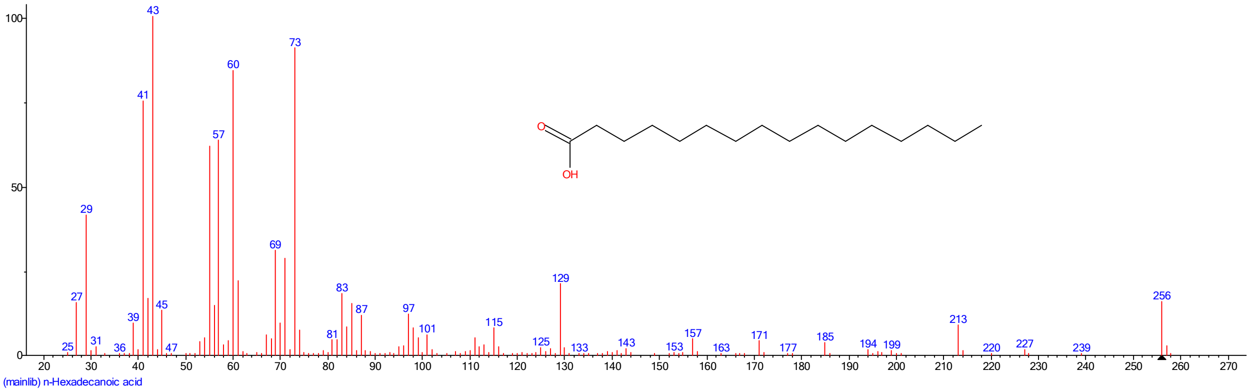

## 21) 3-methyl-1-Butanol,

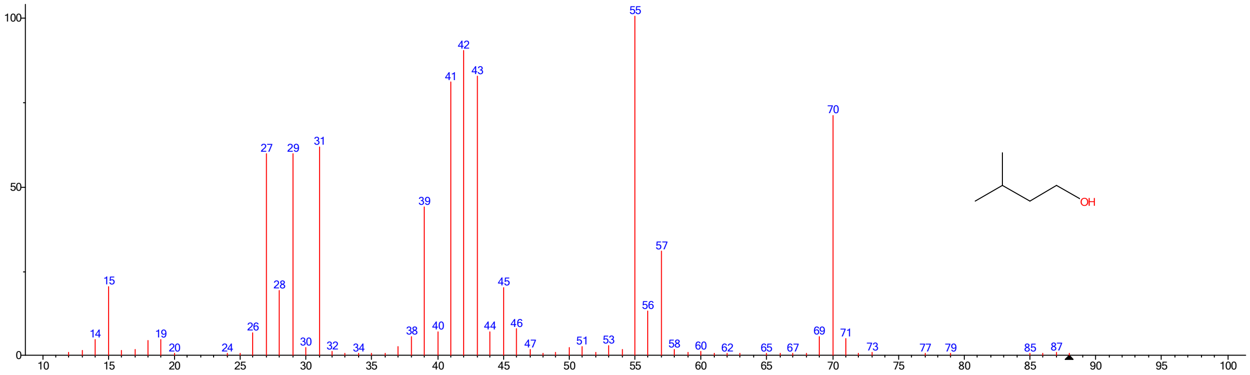


(mainlib) 1-Butanol, 3-methyl-

## 22) 3-methyl-2-Buten-1-ol

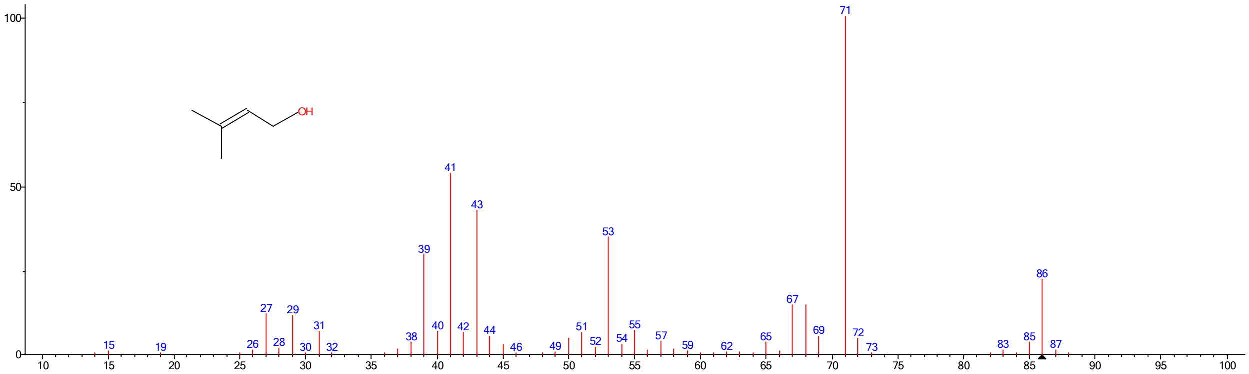


(mainlib) Prenol

## 23) Linalool

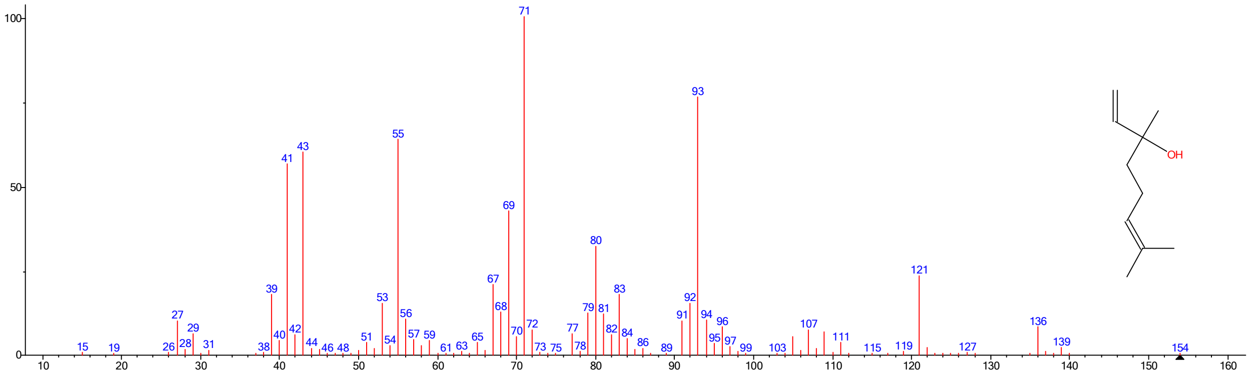


(mainlib) Linalool

## 24) Eugenol

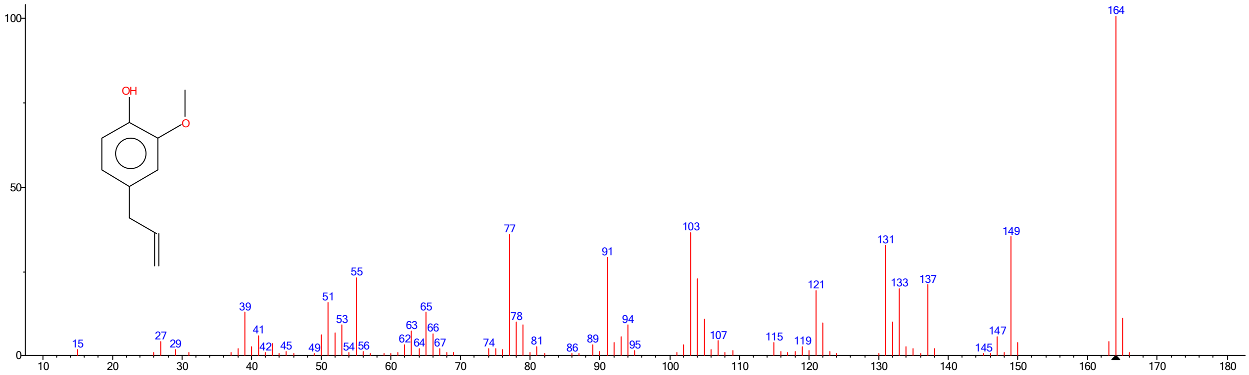


(mainlib) Eugenol

## 25) (R)- 5,6,7,7a-tetrahydro-4,4,7a-trimethyl-2(4H)-Benzofuranone

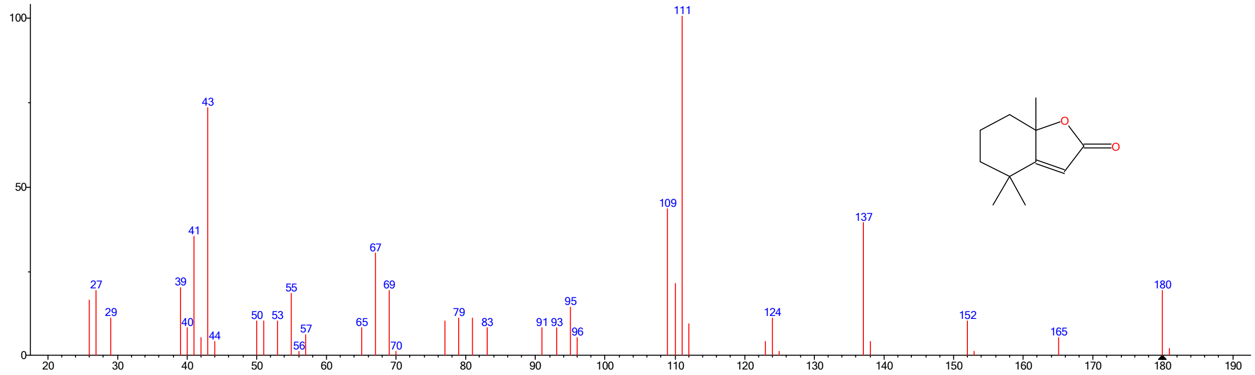


(mainlib) 2(4H)-Benzofuranone, 5,6,7,7a-tetrahydro-4,4,7a-trimethyl-

## 26) Dodecane

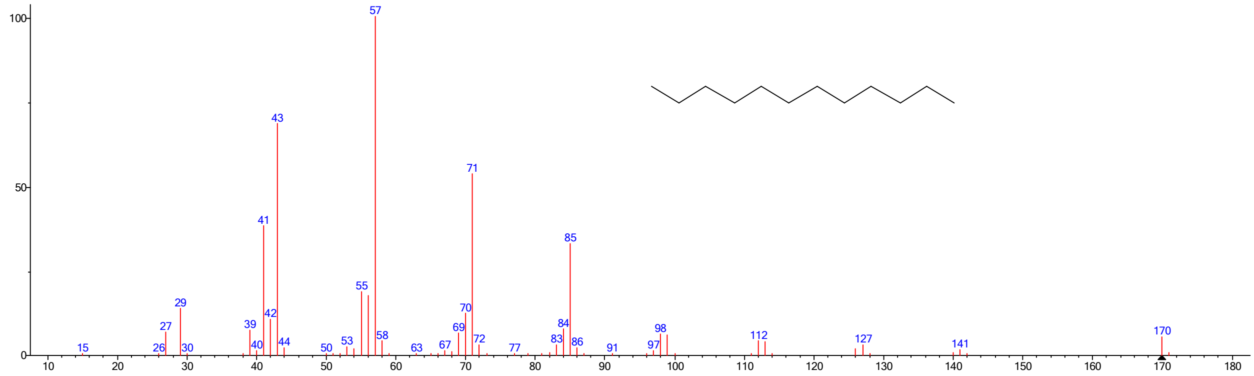


(mainlib) Dodecane

## 27) 1-Dodecane

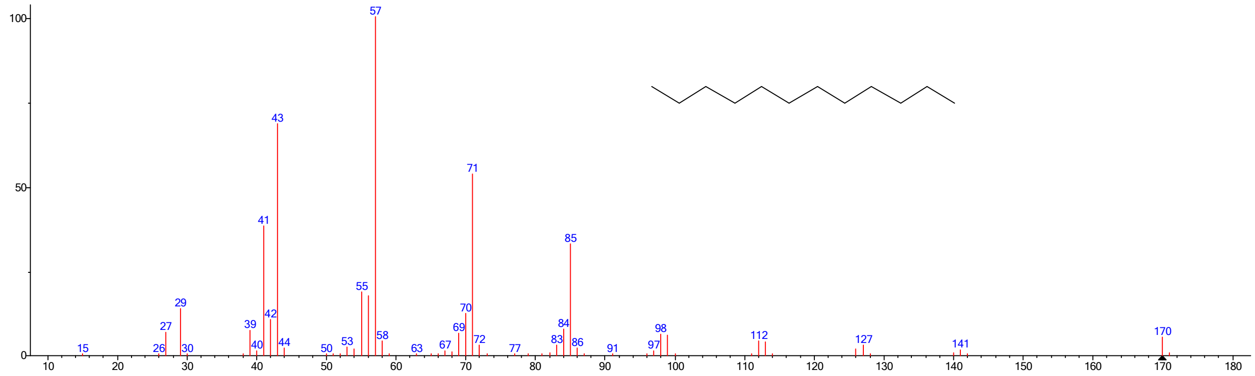


(mainlib) Dodecane

## 28) 1-Tetradecene

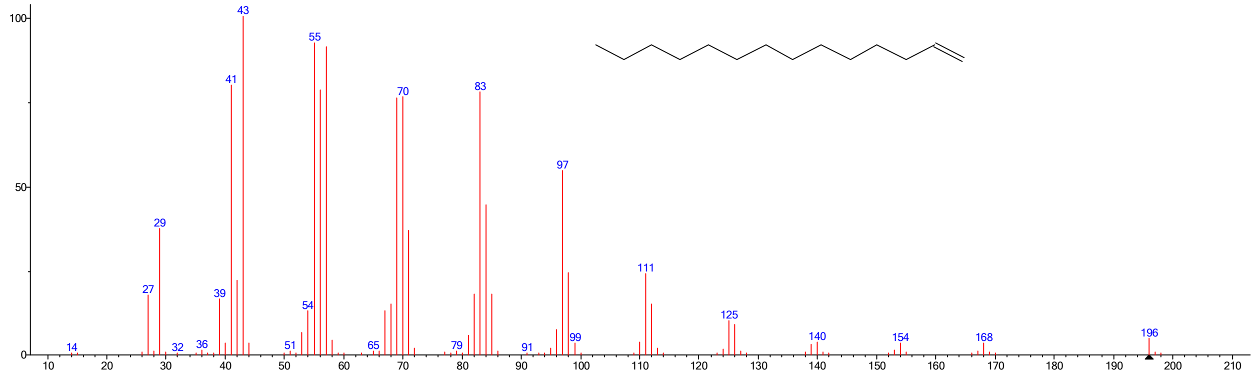


(mainlib) 1-Tetradecene

## 29) Benzeneacetaldehyde

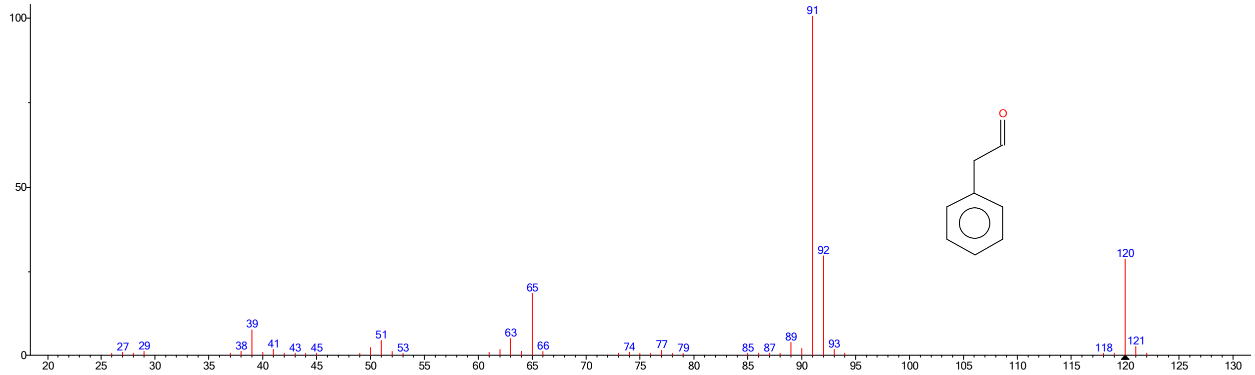


(mainlib) Benzeneacetaldehyde

## 30) Nonadecane

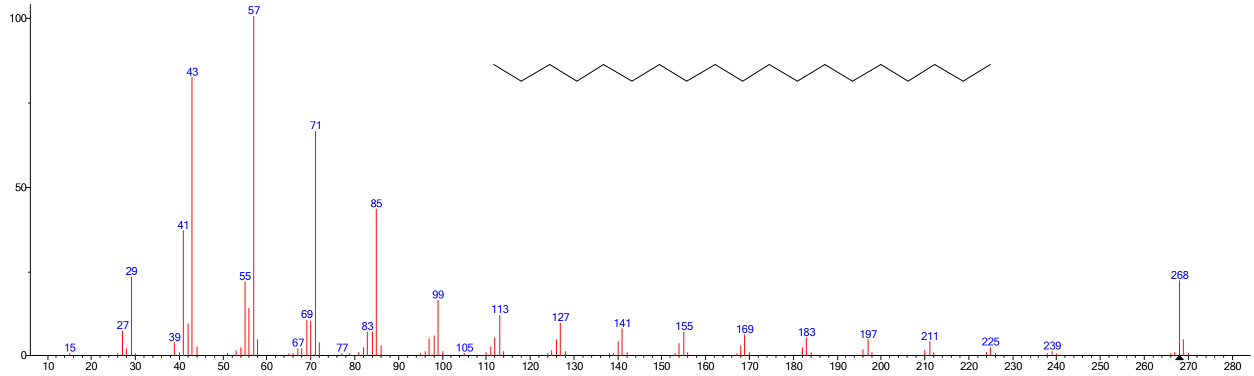


(mainlib) Nonadecane

## 31) Octadecane

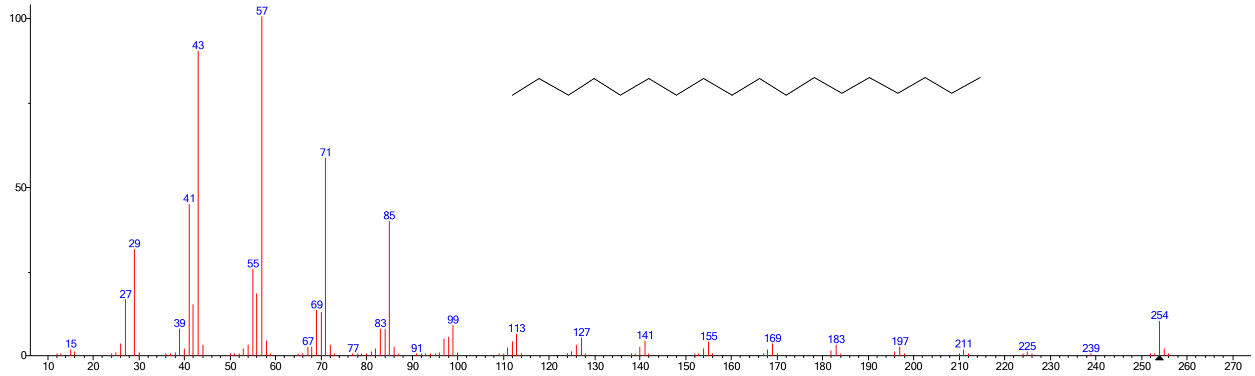


(mainlib) Octadecane

## 32) Hexadecanoic acid

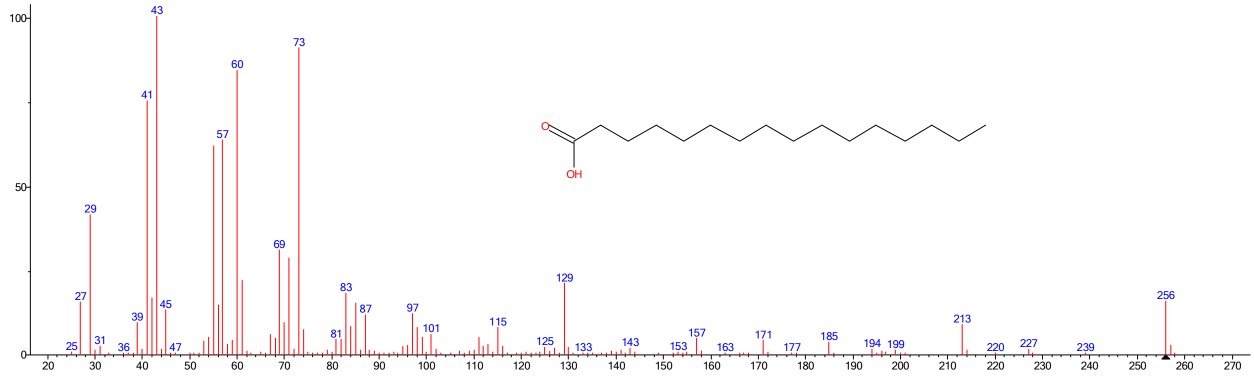


(mainlib) n-Hexadecanoic acid

## 33) Pentadecane

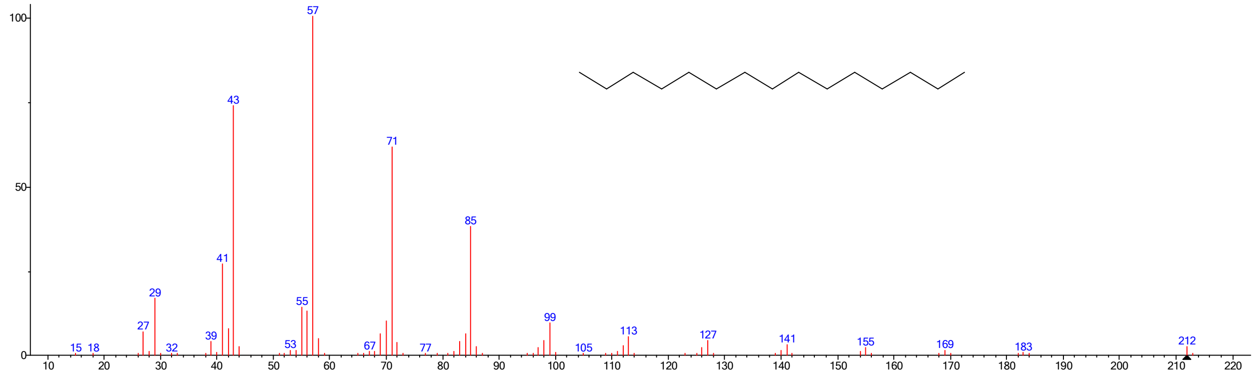


(mainlib) Pentadecane

## 34) Dimethyl Disulfide,

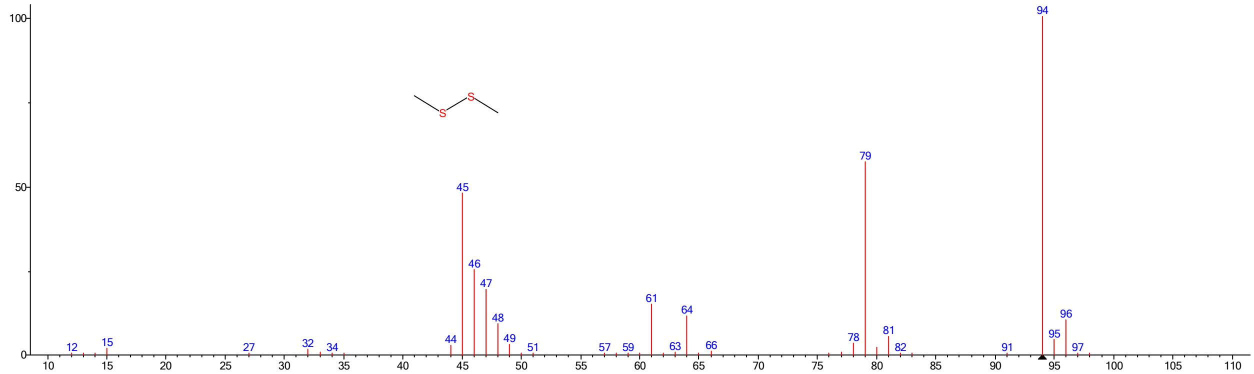


(mainlib) Disulfide, dimethyl

## 35) Hexanoic acid

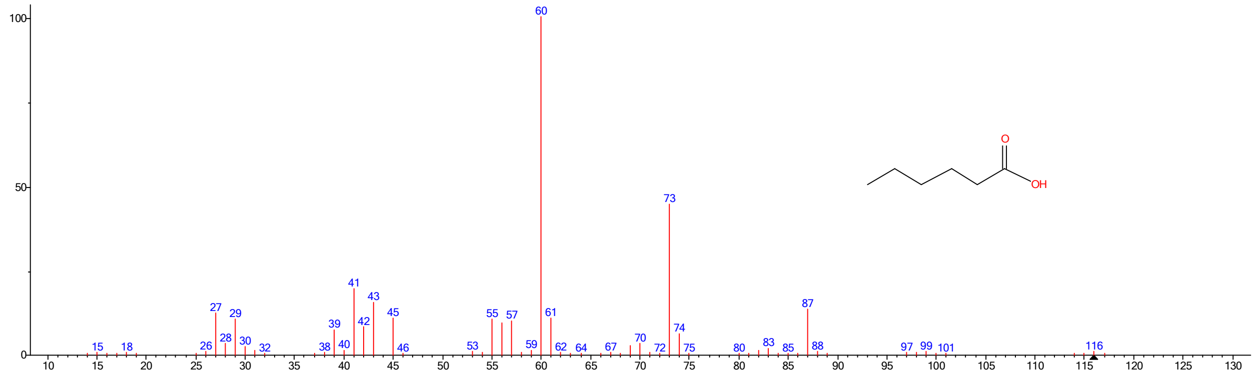


(mainlib) Hexanoic acid

## 36) Octanoic acid

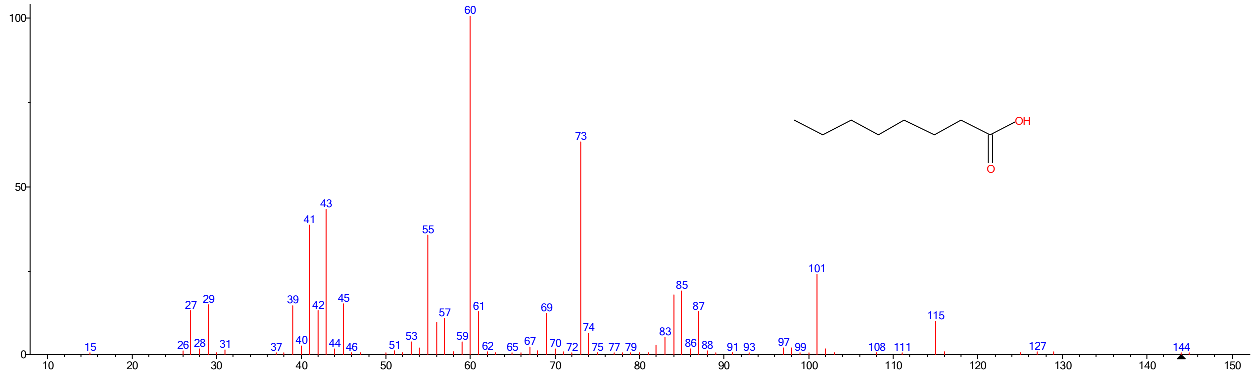


(mainlib) Octanoic acid

## 37) 4-methyl-1-Penten-3-ol

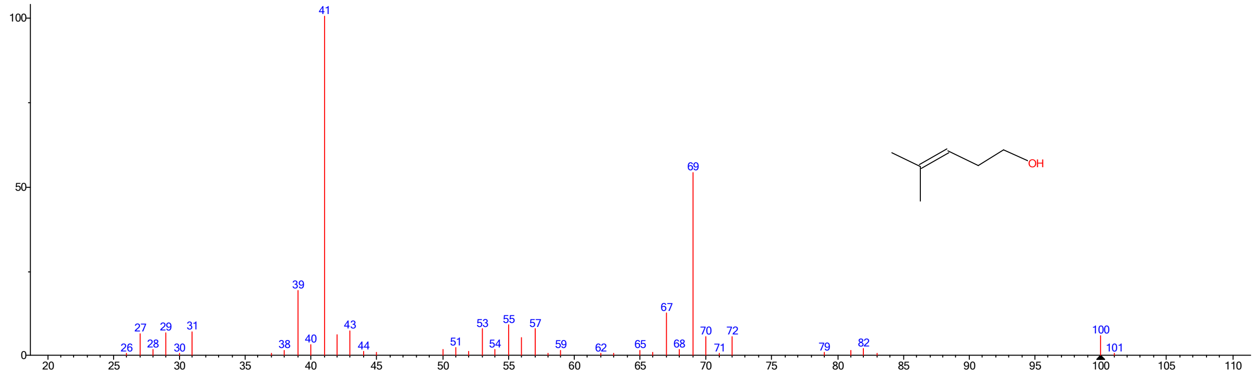


(mainlib) 3-Penten-1-ol, 4-methyl-

## 38) 3-methyl-2-Butenal

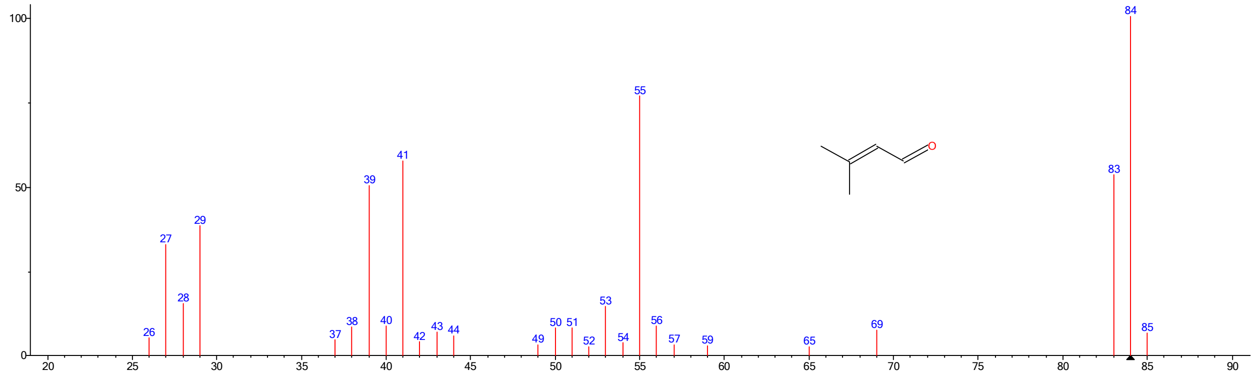


(mainlib) 2-Butenal, 3-methyl-

## 39) 1-Hexen-3-ol

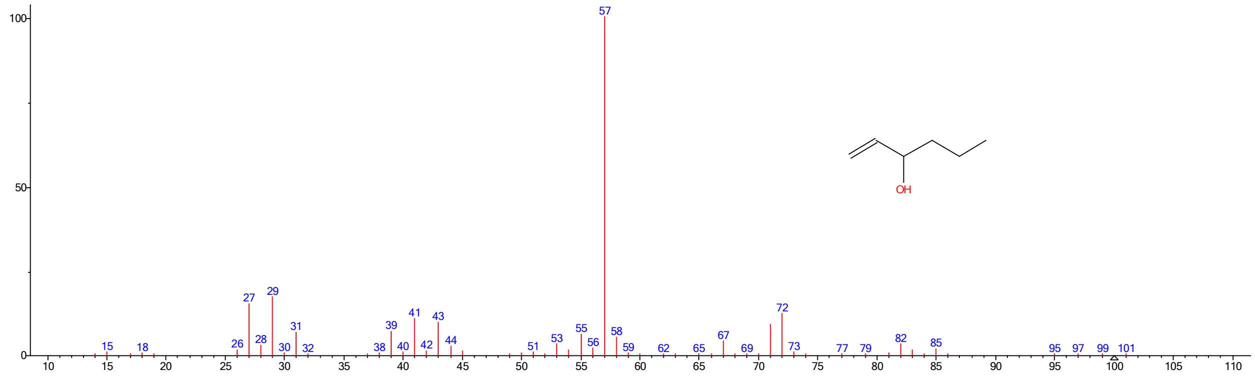


(mainlib) 1-Hexen-3-ol

## 40) 2-Pentanone

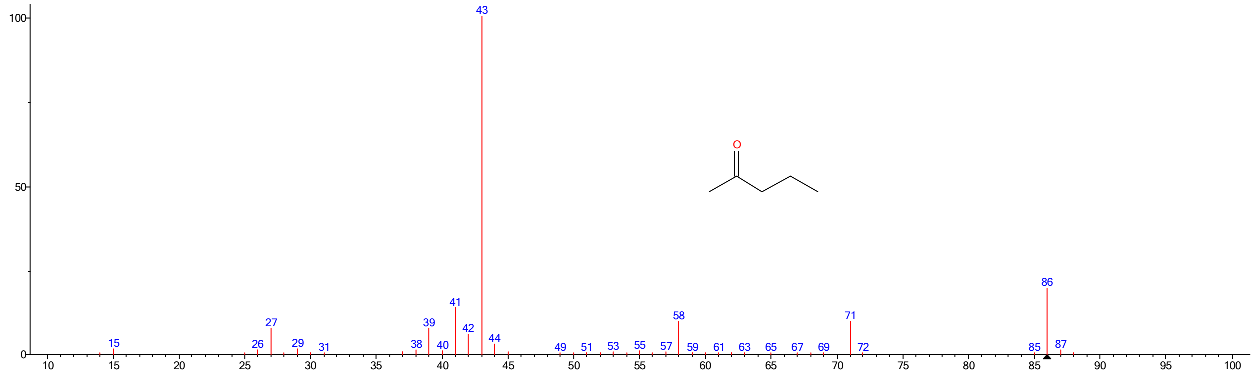


(mainlib) 2-Pentanone

## 41) 2,3,5-trimethyl- Hexane

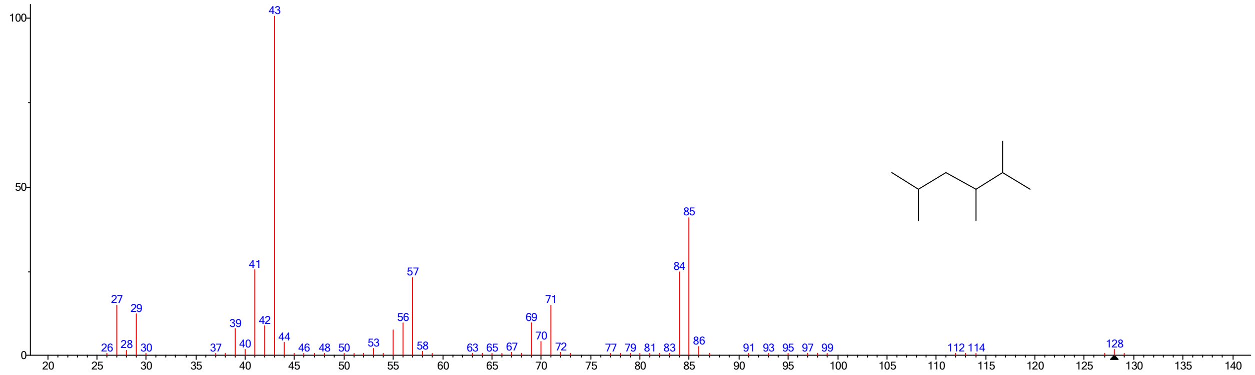


(mainlib) Hexane, 2,3,5-trimethyl-

## 42) 2,3,4-trimethyl- Hexane

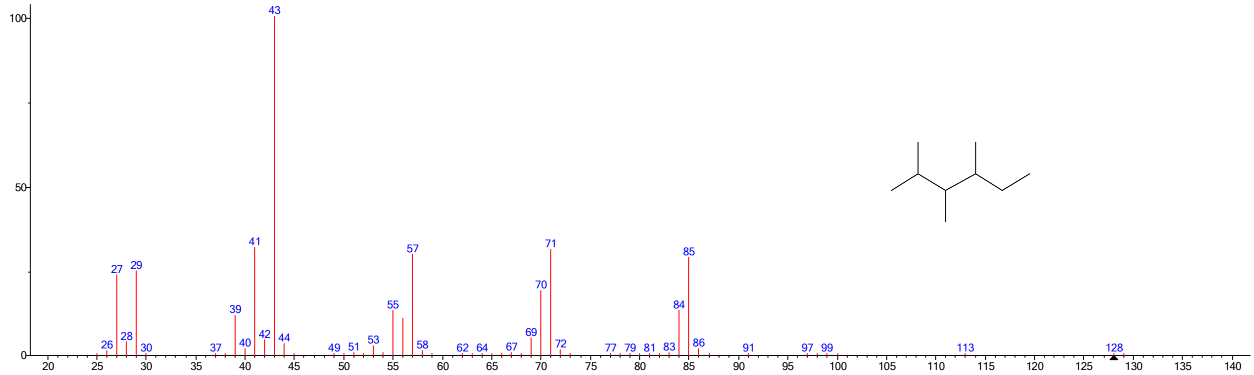


(mainlib) Hexane, 2,3,4-trimethyl-

## 43) 4,4,5-trimethyl-2-Hexene,

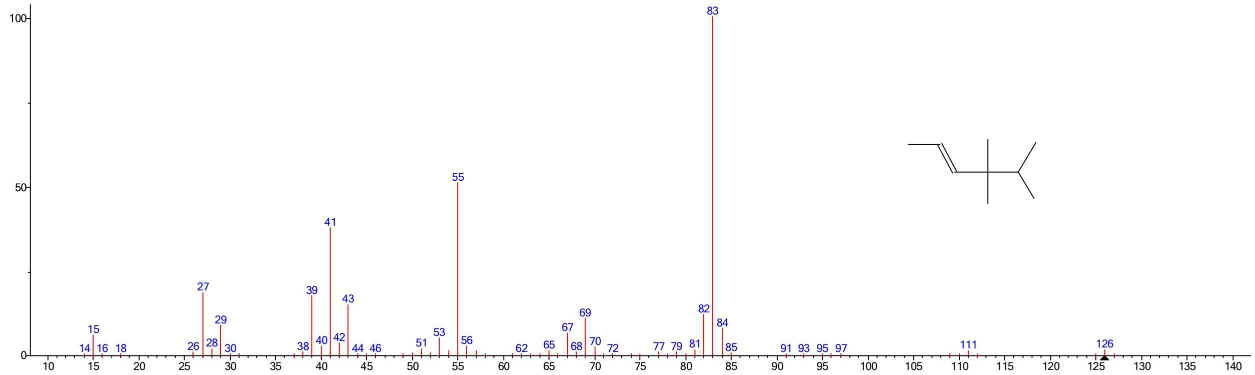


(mainlib) 2-Hexene, 4,4,5-trimethyl-

## 44) 3,5,5-trimethyl-2-Hexene,

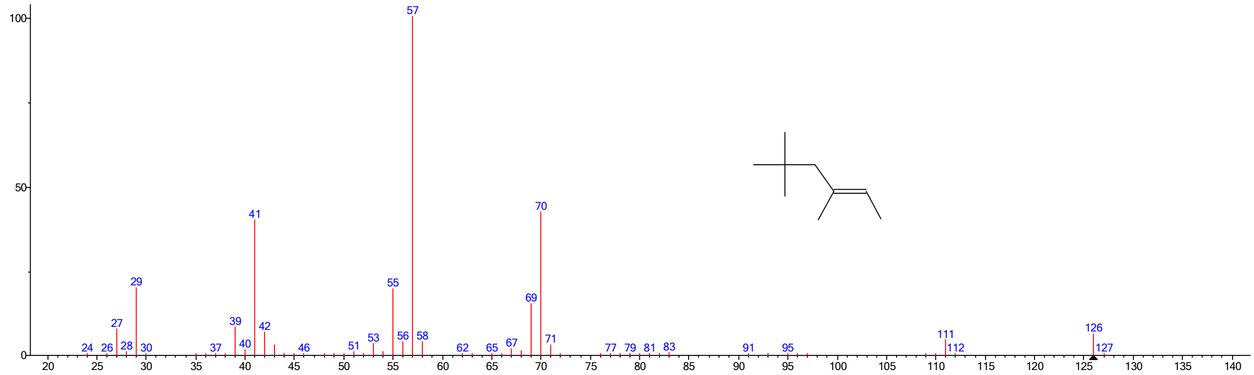


(mainlib) 2-Hexene, 3,5,5-trimethyl-

## 45) 3,5,5-trimethyl-1-Hexene,

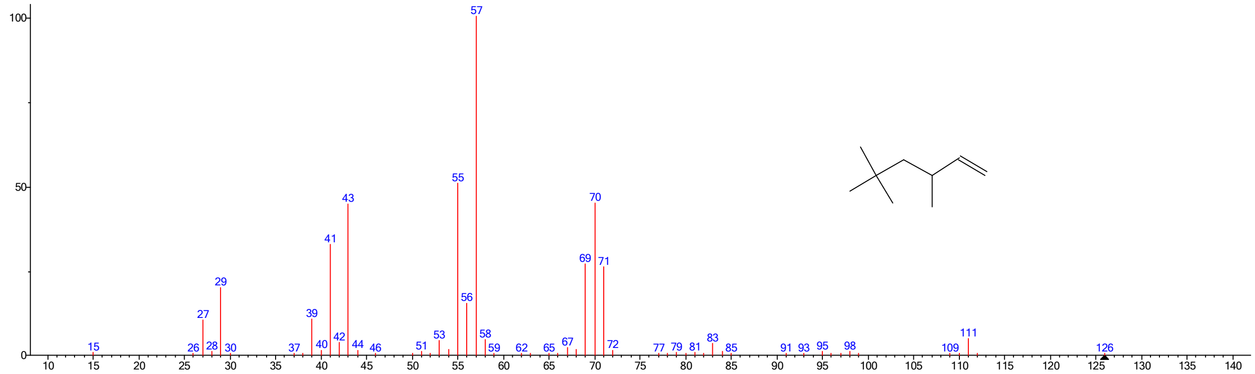


(mainlib) 1-Hexene, 3,5,5-trimethyl-

## 46) 2,3,3-Trimethyl-1-hexene

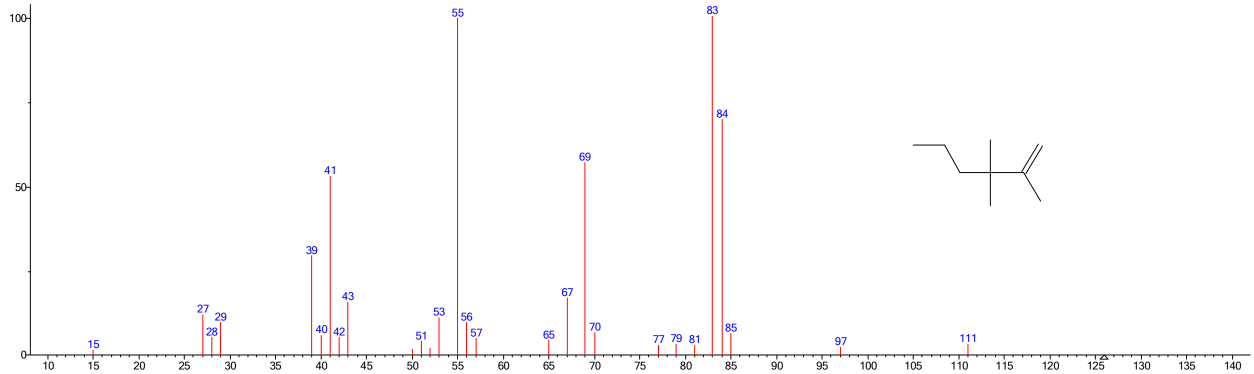


(mainlib) 2,3,3-Trimethyl-1-hexene

## 47) 4,4-dimethyl- Heptane,

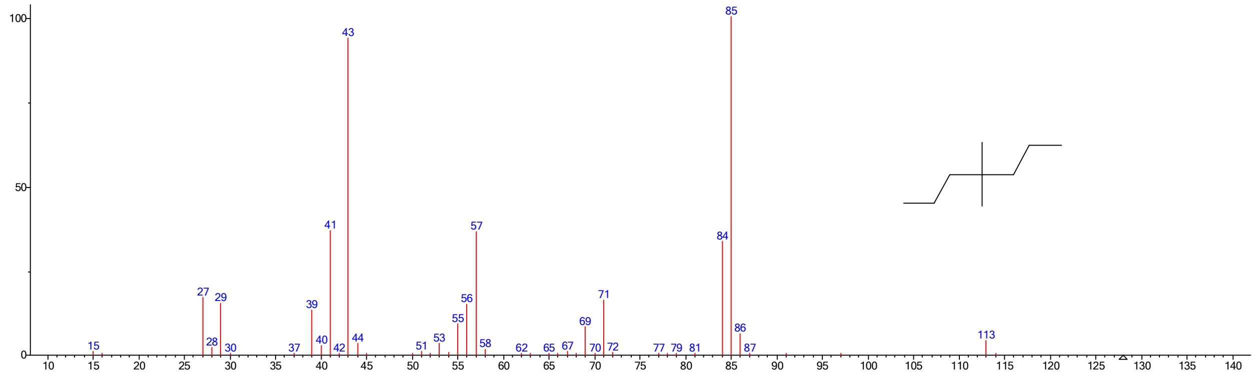


(mainlib) Heptane, 4,4-dimethyl-

## 48) 2,3-dimethyl- Heptane,

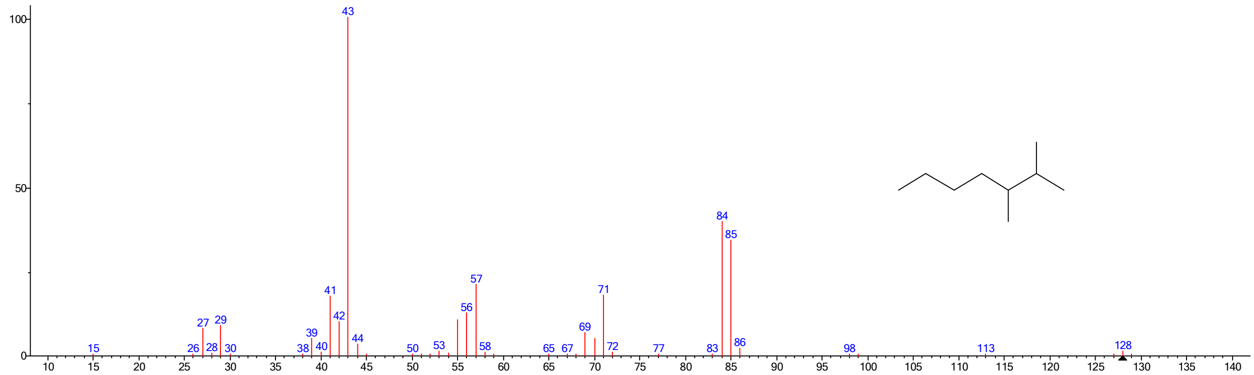


(mainlib) Heptane, 2,3-dimethyl-

## 49) 3-Penten-2-ol

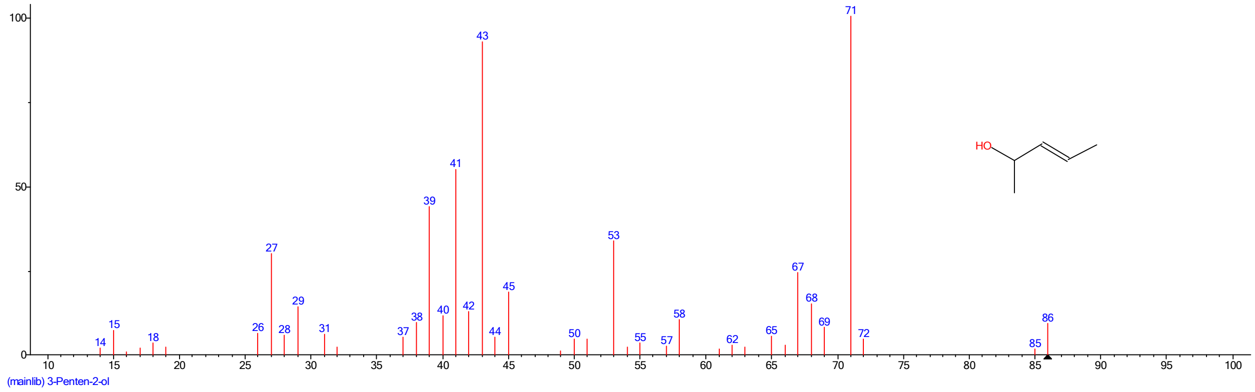


## 50) 3-methyl-3-Buten-1-ol

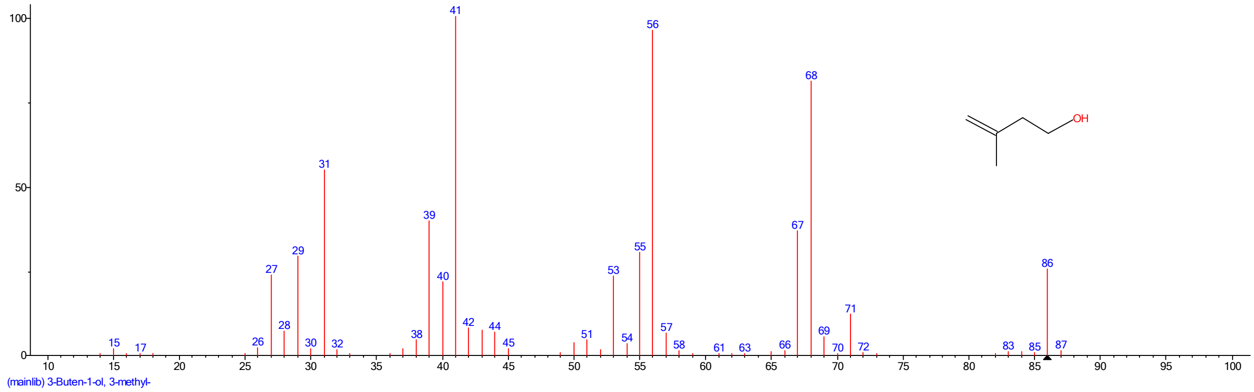


## 51) 2-Octene

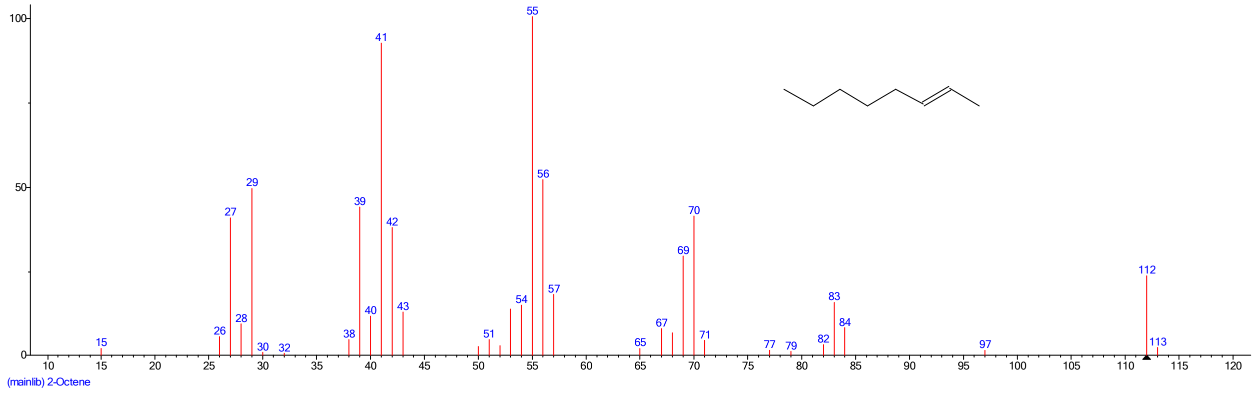


Fig.S.1 GC-TOF of Mass spectrum and structure of compounds
