# Supplementary material for: Development of a Gas Chromatography-Time-of-Flight Method for Detecting Glucosinolate Metabolites and Volatile Organic Compounds in Kimchi
Source: Int J Anal Chem. 2021 Jun 18;2021:9978251. doi: 10.1155/2021/9978251 (PMC8233085; doi:10.1155/2021/9978251)
Supplement: Supplementary Materials — More detailed composition analysis of glucosinolate metabolite and VOC are presented. Table 1: area of glucosinolate metabolites. Table 2: area of volatile oragnic compounds. Figure S1: GC-TOF of mass spectrum and structure of compounds. [file 9978251.f1.zip › 9978251.f1/Supplement Table. 1.pdf]

Supplement Table. 1. Area of Glucosinolate metabolites.

Name	*A	*B	*C	*D	*E	*F	*G	*H	*I	*J	*K	*M
1	-	-	-	57,538	14,668,389	573,153	-	195,649	-	1,820,938	182,944	-
2	-	-	-	60,994	24,227,618	1,067,954	-	466,902	-	3,582,910	501,232	1,068,708
3	-	-	-	69,024	13,095,108	330,661	-	1,196,301	-	1,502,687	172,812	-
4	-	-	-	-	19,552,427	560,073	-	1,513,303	-	2,045,278	579,700	695,234
5	-	-	-	145,203	8,348,032	286,724	-	175,391	-	1,492,805	196,920	-
6	-	-	-	98,561	4,268,012	212,724	-	88,040	-	789,805	728	-
7	-	-	-	137,845	8,920,171	271,375	-	373,398	-	2,730,246	135,781	373,052
8	-	-	-	181,714	8,085,362	326,730	-	384,809	-	2,398,690	180,274	-
9	-	-	-	789,283	15,775,011	874,158	-	3,020,282	-	2,937,743	375,775	1,477,694
10	-	-	-	452,954	11,272,009	402,576	-	1,806,668	-	2,103,644	269,417	949,895
11	-	-	-	-	-	162,684	-	-	-	1,465,316	707,081	-
12	-	-	-	-	-	135,804	-	27,321	-	734,073	267,232	-
13	50,550	-	-	-	-	92,079	-	-	-	467,865	519,558	-
14	53,553	-	-	-	-	63,657	-	23,270	-	434,382	486,403	-
15	3,850,289	-	-	37,171	-	207,150	-	152,123	-	853,095	421,459	3,524,958
16	206,159	-	-	55,332	-	130,807	-	322,714	-	2,593,957	538,597	174,432
17	116,851	416,404	687,574	554,095	16,889,022	37,200,818	14,271,345	7,444,374	1,848,734	2,517,552	81,644	229,507,199
18	153,333	-	-	68,347	-	177,412	-	235,294	-	398,732	611,837	-
19	2,841,538	-	-	36,906	-	183,920	-	152,693	-	914,037	456,690	2,594,569
20	60,806	-	-	-	-	66,179	-	212,488	-	425,829	553,929	-
21	53,687	284,383	731,162	727,574	9,631,148	39,458,110	13,540,570	8,185,858	2,622,951	2,568,594	136,320	278,971,384
22	-	-	-	-	39,724,396	1,405,053	-	906,362	-	6,495,248	862,991	1,671,570
Average	1,385,019	420,472	709,368	248,039	14,958,208	4,140,482	13,905,958	1,521,693	2,683,011	1,876,065	418,949	55,822,360

*A Sulforaphane
*B 1-isothiocyanato heptane
*C Cyclopropylacetonitrile
*D 2-isothiocyanato butane
*E Benzenepropanenitrile
*F 2-isothiocyanatoethyl benzene
*G 5-ethenyl-2-oxazolidinethione
*H 2-(5-Methoxy-2-oxo-1,3-dihydroindol-3-yl)acetonitri
*I 1-Isothiocyanato-6-(methylsulfinyl)hexane
*J 1H-Indole-3-acetonitrile
*K 4-isothiocyanato-1-(methylthio)-1-butene
*M 4-isothiocyanato-1-butene
