# Supplementary material for: Development of a Gas Chromatography-Time-of-Flight Method for Detecting Glucosinolate Metabolites and Volatile Organic Compounds in Kimchi
Source: Int J Anal Chem. 2021 Jun 18;2021:9978251. doi: 10.1155/2021/9978251 (PMC8233085; doi:10.1155/2021/9978251)
Supplement: Supplementary Materials — More detailed composition analysis of glucosinolate metabolite and VOC are presented. Table 1: area of glucosinolate metabolites. Table 2: area of volatile oragnic compounds. Figure S1: GC-TOF of mass spectrum and structure of compounds. [file 9978251.f1.zip › 9978251.f1/Supplement Table. 2.pdf]

Supplement Table. 2. Area of Volatile Oragnic Compounds.

Name	1	2	3	4	5	6	7	8	9	10	11	12	13	14	15	16	17	18	19	20	21	22	average
*A	188,136,937	253,613,243	169,853,163	236,806,476	231,939,688	138,758,906	207,884,940	201,782,070	231,345,723	160,453,268	275,021,365	187,661,547	235,209,783	186,660,798	238,114,795	214,720,467	187,832,042	263,894,944	233,304,774	236,336,394	219,346,083	219,998,077	214,485,249
*B	138,492,761	229,242,315	133,778,289	182,511,813	175,634,213	98,216,092	168,843,830	137,212,791	211,472,827	132,749,870	282,759,784	130,400,281	177,500,846	160,022,776	200,180,630	164,329,134	165,551,262	222,322,764	186,723,029	202,326,484	182,024,042	202,412,724	176,577,662
*C	113,632,437	175,072,101	105,577,050	140,157,961	150,543,832	83,618,997	139,306,126	115,494,728	154,077,690	104,344,582	197,537,056	111,019,555	145,425,821	130,445,216	171,035,716	131,980,033	119,004,603	173,416,968	153,686,427	158,637,589	139,443,688	154,540,256	139,454,474
*D	83,344,146	255,208,994	82,674,439	137,808,801	104,907,267	32,690,648	92,135,595	64,598,477	146,813,974	67,980,608	267,191,864	61,995,992	161,372,366	151,391,178	125,465,717	190,930,578	31,849,714	166,223,035	102,241,141	147,887,697	40,607,486	242,733,122	125,527,724
*E	1,655,340	2,336,075	3,196,637	223,102,672	3,842,407	1,558,264	1,490,590	259,303,653	183,769,034	744,380	251,822,393	974,890	263,916,076	857,725	1,629,052	1,662,297	-	-	-	-	-	-	122,646,591
*F	66,912,650	112,455,893	61,241,706	89,558,266	89,651,842	50,230,196	84,532,056	68,155,780	103,855,296	63,809,833	132,402,677	65,595,027	86,426,236	78,317,865	105,331,262	80,340,700	79,839,031	112,330,137	97,397,876	96,180,026	79,174,260	83,600,313	86,034,155
*G	76,705,672	99,311,758	58,822,015	85,834,371	102,417,569	57,114,720	70,440,725	89,913,054	81,177,004	51,052,041	112,115,279	82,857,606	94,256,522	60,938,044	91,603,739	87,169,815	57,127,174	100,562,582	95,037,117	84,260,542	79,910,066	74,482,263	81,504,986
*H	29,570,764	55,185,887	30,458,090	39,749,249	42,338,951	24,619,173	35,462,173	32,124,526	51,513,715	31,896,289	65,696,049	31,779,711	37,167,301	37,724,323	48,719,314	40,483,713	37,762,184	51,734,898	45,966,579	45,830,900	39,750,132	47,433,470	41,043,972
*I	28,500,433	49,737,453	30,599,197	39,922,523	37,693,874	21,776,630	35,487,268	31,193,153	51,075,623	30,896,744	62,557,688	27,806,413	39,528,642	34,993,994	43,263,513	33,640,296	33,512,921	45,619,277	39,342,463	41,893,398	39,426,417	42,165,643	39,619,137
*J	11,887,456	17,591,479	5,650,838	15,450,727	2,319,663	2,871,485	515,206	12,192,320	22,209,733	9,979,099	67,710,038	42,905,437	34,033,689	26,399,336	35,771,742	32,886,851	529,797	26,344,022	41,883,710	23,987,217	2,513,622	20,674,018	22,441,352
*K	8,288,872	27,972,747	14,502,327	22,213,871	9,885,253	2,758,669	3,739,624	2,425,221	13,135,095	9,963,228	67,965,184	28,646,114	6,613,846	7,299,814	9,466,250	5,166,613	267,725	6,490,966	8,159,390	8,330,693	467,838	22,876,174	13,869,460
*L	2,188,140	2,875,511	1,768,831	29,682,744	2,848,621	1,673,395	1,987,102	30,830,098	73,338,273	1,567,261	49,079,690	2,360,027	2,706,924	1,812,951	2,765,749	2,603,235	1,804,076	3,185,449	3,019,338	2,549,961	2,558,725	2,499,166	10,259,330
*M	1,396,480	3,791,760	11,023,410	16,992,477	2,959,402	1,345,621	10,131,318	8,157,727	11,897,735	9,903,055	5,365,129	2,270,536	5,308,339	4,350,053	16,461,058	4,986,193	44,665	26,388,696	16,702,642	23,459,540	62,465	3,557,441	8,479,806
*N	6,492,265	8,900,728	6,325,575	8,538,159	9,151,796	6,318,224	6,590,801	7,813,830	9,008,998	5,717,997	10,089,914	8,021,929	8,041,299	6,567,580	8,054,864	9,373,896	8,813,219	10,996,457	9,727,199	9,651,644	8,770,012	11,058,904	8,364,786
*O	6,655,539	8,987,615	5,241,579	7,580,954	8,018,966	5,874,991	6,139,096	7,148,033	7,008,730	6,626,801	10,430,563	7,971,993	8,503,912	7,816,924	8,634,057	8,429,141	3,813,132	11,068,973	9,657,366	8,475,692	6,400,704	6,728,950	7,600,623
*P	1,059,252	1,429,689	955,995	17,384,421	1,530,305	909,022	884,260	15,677,436	16,078,131	796,439	19,202,088	1,087,713	16,432,846	842,238	1,347,494	1,237,968	941,032	1,601,589	1,280,696	1,327,818	1,269,265	1,464,489	7,310,528
*Q	6,098,271	8,716,399	-	-	-	-	-	-	-	225,211	-	-	-	-	-	193,634	-	-	232,511	-	-	9,007,322	5,647,696
*R	3,374,411	19,117,948	3,271,998	4,578,799	7,145,802	2,551,572	2,785,341	2,311,040	6,351,153	2,719,600	11,143,432	4,270,431	4,588,225	5,187,287	3,615,288	3,366,055	2,181,438	7,521,589	3,142,470	6,279,669	2,647,904	9,806,319	5,361,717
*S	3,514,426	5,762,173	3,375,326	6,134,349	4,777,253	2,763,887	4,310,877	2,922,251	4,600,764	2,516,625	4,943,102	2,899,262	3,324,125	3,005,053	4,314,237	2,908,874	3,307,414	3,986,782	3,569,873	3,694,312	3,482,347	3,975,755	5,313,708
*T	-	-	-	602,152	-	542,037	-	719,885	1,757,244	-	8,235,603	2,380,121	2,706,240	2,553,435	2,301,578	2,483,607	-	1,394,554	4,893,586	-	-	3,873,985	5,166,604
*U	2,759,655	6,045,631	3,051,956	4,250,845	4,719,380	2,116,351	3,968,597	2,958,241	6,320,937	4,027,106	7,148,480	3,144,132	4,285,114	4,205,682	5,635,521	3,051,905	5,922,864	6,417,337	4,335,602	7,240,590	6,332,754	6,733,191	4,757,812
*V	872,249	3,667,369	956,811	2,213,228	5,845,058	1,338,502	1,232,861	-	3,584,874	911,909	5,450,907	1,107,151	1,888,689	1,684,702	550,336	1,466,237	267,664	1,997,449	-	1,855,495	285,633	7,068,778	4,148,053
*W	1,487,660	2,938,425	2,171,968	3,130,044	2,034,849	1,029,469	1,888,383	1,706,995	3,382,958	2,080,172	11,015,742	4,764,561	5,958,639	5,524,319	6,198,615	7,256,580	-	4,078,650	6,800,603	3,798,199	-	5,240,991	4,124,391
*X	1,765,094	2,822,112	1,564,851	2,103,845	2,984,907	1,121,266	1,981,425	1,592,303	2,559,531	1,567,992	4,827,909	1,462,775	2,063,482	1,924,095	5,582,029	1,841,054	1,930,806	3,532,609	2,178,390	3,521,623	2,101,570	2,570,451	4,099,861
*Y	759,620	6,297,202	963,296	2,567,175	1,749,784	685,768	1,204,133	1,987,715	3,232,922	562,423	2,042,616	1,001,367	725,878	1,242,613	2,070,678	3,960,300	-	2,757,880	3,654,731	2,890,907	1,693,042	2,584,147	3,825,788
*Z	1,560,193	2,095,428	1,859,944	2,754,621	1,979,545	1,326,780	1,551,465	2,342,653	2,859,269	1,690,833	1,905,600	2,423,823	2,877,068	1,615,946	2,334,505	2,070,032	1,042,231	2,482,026	2,473,255	2,163,298	1,807,709	2,195,422	3,679,399
*AA	-	-	-	-	-	-	-	-	554,363	-	-	-	-	-	-	-	498,262	-	-	2,411,313	2,294,789	-	2,879,364
*BB	2,313,581	3,163,880	1,911,235	3,057,720	3,297,001	2,234,667	2,305,259	2,983,944	2,921,985	1,807,783	4,094,199	2,789,640	3,251,632	2,223,094	2,938,978	3,027,720	1,983,284	3,424,398	3,422,799	2,912,297	2,770,180	2,692,513	2,796,718
*CC	1,748,691	3,059,327	3,091,734	4,729,652	1,836,132	802,492	1,393,198	1,137,175	2,958,599	2,215,968	1,661,306	843,339	3,297,434	3,080,181	2,315,844	2,253,204	-	5,745,691	2,181,895	4,940,391	-	4,595,027	2,694,364
*DD	1,617,816	2,583,574	2,209,345	4,241,329	1,462,003	2,871,263	1,670,384	3,983,170	4,353,491	369,504	4,341,942	2,063,475	3,584,052	596,788	1,769,074	2,343,578	1,569,414	1,527,015	1,717,893	1,140,153	3,585,661	4,215,279	2,646,698
*EE	2,208,244	3,436,222	1,418,254	1,517,011	2,260,052	1,322,177	907,994	1,107,940	1,523,404	1,450,312	4,894,066	1,919,568	2,708,731	2,134,705	1,577,236	2,592,056	-	2,072,858	1,657,608	1,883,734	-	4,581,001	2,354,900
*FF	1,197,708	1,908,817	960,219	1,116,124	2,006,039	1,644,762	1,172,680	936,499	2,097,011	1,075,586	2,234,688	965,999	651,344	831,389	1,105,750	880,347	1,503,125	1,775,269	1,159,861	2,065,216	1,600,769	498,701	2,057,925
*GG	1,827,366	2,338,361	1,428,463	1,893,855	2,319,439	1,399,670	2,120,144	1,619,646	2,053,638	1,296,453	2,515,147	1,579,001	1,967,928	1,766,436	2,655,432	2,116,267	1,746,388	3,090,146	2,307,967	2,338,184	2,125,337	2,228,943	2,033,373
*HH	700,030	481,861	524,974	842,853	3,531,402	1,661,062	997,551	655,297	886,601	379,511	9,404,777	4,502,673	1,023,290	1,423,777	2,382,650	-	261,928	1,129,776	1,599,323	1,414,173	285,305	697,115	1,768,776
*II	1,170,600	1,609,802	801,726	1,384,026	300,482	150,135	483,311	600,186	1,162,039	868,746	1,758,698	818,760	2,093,966	1,451,436	6,630,892	1,092,497	-	836,864	6,052,502	681,777	-	1,882,648	1,591,555
*JJ	930,814	1,371,491	2,314,288	4,663,125	787,768	98,640	1,672,887	723,127	892,927	665,728	884,782	149,685	191,011	-	-	1,238,264	-	1,032,437	-	922,373	-	871,028	1,573,814
*KK	1,015,060	1,550,290	1,609,193	4,157,797	554,758	-	1,475,602	2,156,803	1,898,060	749,460	360,999	217,689	291,264	352,421	-	1,709,295	-	951,919	-	825,284	-	668,845	1,208,514
*LL	1,042,289	2,275,483	1,373,228	430,838	1,969,447	1,046,709	1,700,597	341,272	406,903	-	723,051	-	426,057	-	-	-	-	-	-	-	-	-	1,100,238
*MM	273,086	853,262	-	57,337	-	-	-	-	-	-	145,444	-	37,442	-	-	-	-	-	-	-	-	1,624,666	897,371
*NN	-	-	-	737,699	-	-	-	642,267	859,821	-	1,295,787	-	773,389	-	-	-	-	-	-	-	-	-	861,793
*OO	385,934	716,645	1,445,862	2,316,617	230,307	48,028	898,539	588,590	997,587	693,958	947,169	363,489	555,402	470,145	641,664	1,288,971	-	805,222	711,248	813,775	-	537,117	799,462
*PP	699,034	2,725,576	50,024	101,463	244,504	30,225	58,495	42,855	175,203	-	-	-	660,458	661,128	135,070	118,770	-	263,799	54,883	191,401	-	1,536,771	645,805
*QQ	-	-	-	-	-	-	-	-	452,143	-	1,048,051	-	459,006	-	-	70,223	-	-	-	-	-	-	608,827
*RR	444,757	646,870	764,075	1,635,242	158,121	-	729,525	598,779	588,888	567,993	189,723	249,440	256,556	327,560	-	774,556	-	848,720	-	458,144	-	323,121	597,629
*SS	282,901	171,144	153,989	457,361	-	496,602	399,504	258,931	381,087	2,423	-	9,410	-	47,699	-	223,837	25,897	-	-	-	-	-	513,668
*TT	270,324	829,815	291,217	392,261	353,675	200,589	348,722	280,997	738,681	316,804	1,031,274	268,206	359,545	364,822	462,201	352,645	398,661	523,154	459,566	789,740	414,126	1,267,458	487,022
*UU	26,829	92,891	59,167	85,034	35,590	23,613	32,363	10,476	48,554	20,828	3,083,100	1,306,362	731,331	678,614	119,137	964,837	-	217,798	-	3,275	-	446	443,544
*VV	35,175	27,267	-	640,399	24,069	14,319	-	551,252	660,236	-	818,886	50,822	603,108	-	61,259	22,877	-	49,382	44,958	24,987	22,149	78,727	294,464
*WW	-	-	-	259,632	-	-	-	211,499	265,741	-	352,845	-	208,067	-	21,206	32,347	15,284	-	-	49,419	36,968	-	290,602
*XX	-	-	-	-	16,860	-	-	-	29,197	-	72,346	42,367	408,217	388,919	-	346,310	-	343,040	-	309,936	-	78,355	265,506
*YY	129,910	262,861	409,107	651,827	152,167	57,024	318,459	237,877	312,980	219,982	21,393	-	128,794	183,061	-	332,056	-	196,943	-	179,128	-	325,537	252,190
*ZZ	180	5,549	28,254	49,333	-	-	3,439	1,410	9,478	5,091	435,271	173,005	339,825	234,112	333,781	296,840	4,639	253,458	351,972	249,739	5,067	138,074	145,926

*A 2,3,4-trimethyl hexane
*B 4,6-dimethyl dodecane
*C 1,3-bis(1,1-dimethylethyl) benzene
*D n-hexadecanoic acid
*E 3,5,5-trimethyl-2-hexene
*F dodecane
*G 2,4-dimethyl-heptane
*H tetradecane
*I pentadecane
*J 3-penten-2-ol
*K hexanoic acid
*L 4,4,5-trimethyl-2-hexene
*M diallyl disulphide
*N 3-methyl-3-buten-1-ol
*O 2-pentanone
*P 2,3,3-Trimethyl-1-hexene
*Q 3,5,5-trimethyl-1-hexene
*R octanoic acid
*S nonadecane
*T 1-hexen-3-ol
*U hexadecane
*V iso-valeric acid
*W (R)-5,6,7,7a-tetrahydro-4,4,7a-trimethyl-2(4H)-benzofuranon
*X octadecane
*Y butanoic acid
*Z 3-methyl-2-butenal

*AA 1-penten-3-one
*BB 3-methyl-1-butanol
*CC methyl 2-propenyl trisulfide
*DD 2,3,5-trimethyl-hexane
*EE benzeneacetaldehyde
*FF 2-methyl-butanoic acid
*GG 2,3-dimethyl-heptane
*HH 1-dodecene
*II dimethyl disulfide
*JJ sabinene
*KK camphene
*LL cis-3-hexenal
*MM 4,4-dimethyl-heptane
*NN 2-methyl-2-butenal
*OO sesquiphellandrene
*PP dimethyl tetrasulphide
*QQ 1-tetradecene
*RR (-)-pinene
*SS linalool
*TT (Z)- 7-methyl-5-undecene
*UU hexadecanoic acid
*VV 2-octene
*WW 3-methyl-2-buten-1-ol
*XX eugenol
*YY farnesene
*ZZ 4-methyl-1-penten-3-ol
